# Dynamic Interfacial Stability Confirmed by Microscopic Optical Operando Experiments Enables High‐Retention‐Rate Anode‐Free Na Metal Full Cells

**DOI:** 10.1002/advs.202005006

**Published:** 2021-05-03

**Authors:** Bingyuan Ma, Youngju Lee, Peng Bai

**Affiliations:** ^1^ Department of Energy, Environmental & Chemical Engineering Washington University in St. Louis St. Louis MO 63130 USA; ^2^ Institute of Materials Science and Engineering Washington University in St. Louis St. Louis MO 63130 USA

**Keywords:** battery safety, impedance diagnosis, interfacial stability, metal anodes, operando microscopy

## Abstract

Rechargeable alkali metal anodes hold the promise to significantly increase the energy density of current battery technologies. But they are plagued by dendritic growths and solid‐electrolyte interphase (SEI) layers that undermine the battery safety and cycle life. Here, a non‐porous ingot‐type sodium (Na) metal growth with self‐modulated shiny‐smooth interfaces is reported for the first time. The Na metal anode can be cycled reversibly, without forming whiskers, mosses, gas bubbles, or disconnected metal particles that are usually observed in other studies. The ideal interfacial stability confirmed in the microcapillary cells is the key to enable anode‐free Na metal full cells with a capacity retention rate of 99.93% per cycle, superior to available anode‐free Na and Li batteries using liquid electrolytes. Contradictory to the common beliefs established around alkali metal anodes, there is no repeated SEI formation on or within the sodium anode, supported by the X‐ray photoelectron spectroscopy elemental depth profile analyses, electrochemical impedance spectroscopy diagnosis, and microscopic imaging.

## Graphical abstract

The ingot‐type Na metal plating and stripping observed in microcapillary diagnostic cells reveals the ideal dynamic interfacial stability, which guarantees the success of anode‐free Na metal full cells.

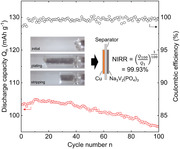

## 1 Introduction

Sodium (Na), as one of the most abundant elements on earth,^[^ ^1^ ^]^ has been considered a sustainable alternative to lithium (Li) for high‐performance and low‐cost rechargeable batteries.^[^ ^2^ ^]^ The physical and chemical similarities between these two alkali metals have already inspired promising developments and improvements of Na‐ion and room‐temperature Na‐metal batteries, following the successes of the Li counterparts. Na‐metal anodes, without the bulky host structure for ion intercalation, could enable batteries with theoretical specific energies as high as 3 to 4 times that of existing Li‐ion batteries.^[^ ^3^ ^]^ However, due to the low redox potential of Na metal, components of the Na‐salt non‐aqueous electrolytes unavoidably get reduced to form a solid electrolyte interphase (SEI) layer that can passivate the Na metal from further reducing the electrolyte, while allowing Na ion to diffuse across and complete the electroplating process.^[^ ^4^, ^5^, ^6^, ^7^, ^8^ ^]^ The irreversible SEI layers have been found to quickly accumulate during battery cycling due to the repeated exposure of fresh surfaces of Na metal, leading to low Coulombic efficiency and short cycle life. It may also induce heterogeneous ionic flux and surface tension that promote dendritic growths to result in internal shorts.^[^ ^2^, ^9^, ^10^ ^]^

Various innovations have been made to achieve stable practical sodium metal anodes,^[^ ^9^ ^]^ including artificial SEI,^[^ ^11^, ^12^, ^13^ ^]^ nanocarbon nucleation layer,^[^ ^14^, ^15^ ^]^ scaffold‐like current collectors,^[^ ^16^, ^17^, ^18^ ^]^ and solid‐state electrolytes.^[^ ^19^, ^20^ ^]^ While very thin dendritic Na filaments can be avoided, postmortem analyses of cycled Na metal cells always show clear surface roughness,^[^ ^3^, ^21^ ^]^ resembling the morphology of Li whiskers of different widths.^[^ ^22^, ^23^ ^]^ Among these approaches, the simple glyme‐based electrolytes,^[^ ^24^ ^]^ first tested for Na metal anodes by Seh et al.,^[^ ^3^ ^]^ appear to enable the best interfacial stability. However, most recent reports of Na metal cells adopting glyme‐based electrolytes either have excess Na metal,^[^ ^25^, ^26^, ^27^ ^]^ or use complex current collectors.^[^ ^14^, ^15^, ^28^, ^29^ ^]^ The capacity retention rate of anode‐free Na metal full cells with only the plain copper current collector on the anode side is still not satisfactory.^[^ ^30^, ^31^ ^]^ All these results indicate that interfacial stability needs precision diagnosis to identify possible improvement strategies. On the other hand, most recent in situ and operando studies of Na electroplating frequently observed whisker‐like^[^ ^8^, ^32^, ^33^ ^]^ or moss‐like^[^ ^34^, ^35^, ^36^, ^37^, ^38^, ^39^, ^40^ ^]^ porous Na structures, accompanied by suspicious gas evolution.^[^ ^32^, ^33^, ^40^, ^41^, ^42^ ^]^

Here, we report for the first time, the self‐modulated shiny‐smooth non‐porous Na metal deposit grown from unpolished rough surfaces, in glyme‐based liquid electrolytes. This ideal interfacial stability confirmed in our *operando* microcapillary cell experiments enables anode‐free full cells that can deliver > 93.4% of the initial capacity after 100 cycles of galvanostatic cycling at 3C (equivalent to 0.75 mA cm^−2^), yielding a Na inventory retention rate (NIRR)^[^ ^31^ ^]^ of 99.93% per cycle. While controlling the moisture level of the electrolyte is critically important, the obtained superior performance is attributed to the lack of repeated formation and accumulation of SEI layers on Na metal deposits, corroborated by the nearly invariable interfacial impedance, and reflected by the high efficiencies in both half and full cells. The comparative electrochemical impedance spectroscopy (EIS) analyses of half and full cells during cycling proved that the cathode electrolyte interphase (CEI) layers, rather than SEIs on the anode, dominate the cell degradation.

## 2 Results

### 2.1 Self‐Modulated Shiny‐Smooth Na Ingots

Glass capillary cells^[^ ^22^ ^]^ were fabricated to investigate the growth mechanisms of Na metal anode, which allow not only the straightforward observation of the morphological evolution, but also the accurate determinations of the actual current density and characteristic physical constants. It is worth noting that according to the definition of electroplating, the working electrode should be called a cathode. However, following the naming convention of batteries, we designated the Na electrode as anode. The cathodic electroplating process occurs on the Na metal “anode” during the battery recharge process.

**Figure 1:**
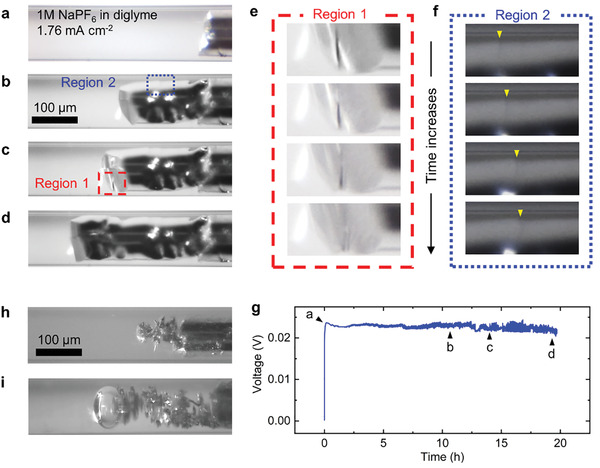
Operando observation of Na electroplating in 1 m NaPF_6_‐diglyme electrolyte within the capillary cell at an under‐limiting current density. a–d,g) Snapshots and the corresponding voltage response of the Na metal plating process, revealing a monolithic piece of shiny smooth Na ingot. e) The wrinkle‐flattening process on the front facet facing the counter electrode. f) The step‐moving process along the side of the Na ingot. h,i) Non‐ideal interfacial stabilities revealed by (h) the mossy growth and (i) a pico‐liter‐sized gas bubble in 1 m NaPF_6_‐diglyme electrolyte but at moisture levels higher than 10 ppm.

In contrast to the highly porous Na deposits^[^ ^8^, ^32^, ^33^, ^34^, ^35^, ^36^, ^37^, ^38^, ^39^, ^40^ ^]^ and Li deposits^[^ ^22^, ^43^, ^44^ ^]^ reported in the literatures, the snapshots shown in **Figure** **1**a–d clearly reveal an ingot of Na, that is, a non‐porous solid piece of Na metal with shiny‐smooth surfaces, “filling up” the capillary. See also Video S1 and Figure S1, Supporting Information. It is worth noting that the initial surface of the electrode has clear deep crevices, but the growth self‐modulates itself into a shiny‐smooth monolithic piece. In addition to the macroscopic smooth facets that can easily reflect the illumination, two microscopic characteristic growth processes were identified. The first is the wrinkle‐flattening process on the front facet facing the counter electrode (Figure 1e), and the other is the step‐moving process along the side of the ingot (Figure 1f). The observed classical layer‐by‐layer step‐moving mechanism clearly supports the formation of a smooth Na ingot.

It is important to note that Na is more sensitive to the trace amount of water in the nonaqueous electrolytes than Li is. In our experiments, the moisture level of the electrolyte must be lower than 10 ppm to consistently get the ingot‐type shiny‐smooth non‐porous Na metal growths. The ideal interfacial stability, reflected by the ingot‐type growths, disappears when the moisture level in the same NaPF_6_‐diglyme electrolyte is slightly increased (Figure 1h). Pico‐liter‐sized gas bubbles that cannot be detected by other methods emerge when the moisture level is slightly higher, especially after galvanostatic cycling (Figure 1i). While a systematic investigation of the effects of trace amount of moisture and the reaction pathways of gas evolution is important and necessary, in the present work, we focus on the assessment of the system with the ideal interfacial stability to demonstrate the possibility of high‐retention‐rate anode‐free full cells, which is of great practical importance.

Even under the careful control of the moisture level, this ingot‐type Na deposit was only observed in electrolytes of specific combinations of salt and solvent. Replacing NaPF_6_ with NaClO_4_ leads to similar whisker‐free Na deposits, but with a much rougher side surface (Figure S2, Supporting Information). Changing the solvent from glymes to typical carbonate mixtures (e.g., ethylene carbonate and dimethyl carbonate widely used in Li‐salt electrolytes) clearly promotes Na whisker/moss growths, regardless of the type of Na salts (Figures S3–S5, Supporting Information). The drastically different morphologies appear to suggest that, the SEI layers formed on Na in the NaPF_6_‐glyme electrolytes did not follow the current wisdom established on the SEIs formed on alkali metal anodes in ether‐ or carbonate‐based electrolytes.^[^ ^45^, ^46^ ^]^ From the physical perspective, the SEI layers formed in NaPF_6_‐diglyme electrolyte must be ionically and electrically “thin” enough that both Na ions and electrons are readily available everywhere to enable the step‐moving growth mechanism observed here (Figure 1), which was never observed in Li electroplating, nor in Na plating in other electrolytes. Yet, the thin SEI layers must be either flexible enough that no mechanical stress would be built up underneath, or strong enough that no pinholes would be created in the SEI layers, such that the “soft” Na metal cannot be “squeezed out” as root‐growing whiskers.^[^ ^22^, ^47^, ^48^ ^]^ The surprising observations appear to suggest yet another possibility that no SEI layers were accumulated on the Na metal surface, just as no SEI coverages would form on the compact metal deposits obtained in aqueous solutions, in which the classic step‐moving mechanism was established.^[^ ^49^, ^50^, ^51^ ^]^

Due to the low redox potentials, the chemical passivation of alkali metal anodes in nonaqueous electrolytes occurs spontaneously, without the need of passing the current. To allow the chemical reaction to prevail, interrupting the Na plating process with long‐time rests, instead of keeping the system in an all‐time dynamic electrochemical process, may allow the SEI layers to grow thicker or evolve into a more stable state, after which the morphology of Na deposits may become different. To test this possibility, transparent capillary cells were fabricated to perform one‐way electroplating with rests. After a plating process at 1.85 mA cm^−2^ for 10 h, the capillary cell was put into rest for 8 h, a typical resting time for cellphone or electric car batteries, that is, during idling or parking. When the plating was resumed with the same current density, the new growth simply continued the step‐moving process from where it had been stopped, without creating any visible marks on the side surface of the shiny‐smooth Na ingot (Video S2, Supporting Information). No visible marks were found even after a second rest of 16 h.

### 2.2 Visual Inspections of the SEI Accumulation

**Figure 2:**
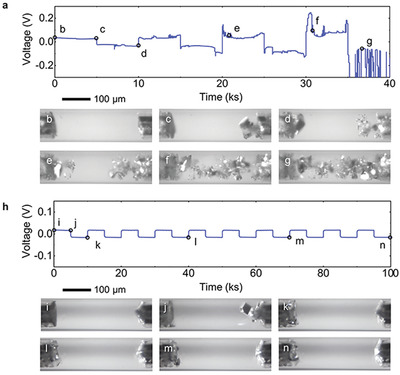
Operando observations of SEI accumulation during cycling at an under‐limiting current density. a–g) 1 m NaClO_4_‐diglyme electrolyte. h–n) 1 m NaPF_6_‐diglyme electrolyte. Both cases were cycled at 2.23 mA cm^−2^ with an areal capacity of 3.10 mAh cm^−2^, that is, 10 000 s per cycle. See also a synchronous comparison of these two experiments in Video S3, Supporting Information.

Our transparent capillary cells offer the direct test of whether inactive SEI layers will trap Na particles and accumulate during plating‐stripping cycling. Symmetrical Na|Na capillary cells with an inter‐electrode separation of ≈380 µm were fabricated and cycled at a constant under‐limiting current density.^[^ ^22^, ^52^ ^]^ As shown in **Figure** **2**, while the cells using 1 m NaClO_4_‐diglyme electrolyte can form similarly smooth Na deposits during the first plating step, some transparent structures (SEI sleeves/gloves) with trapped shiny Na particles were left in the bulk electrolyte after the completion of the first stripping step (Figure 2a–g, Video S3, Supporting Information). The structures quickly accumulate on both electrodes upon cycling, leading to an interpenetrating structure and large voltage fluctuations. The results clearly explain the low Coulombic efficiency of this chemistry,^[^ ^3^ ^]^ and are consistent with the prevailing belief that repeated formation of SEI layers on newly exposed metal surfaces continuously reduce the electrolyte and trap disconnected metals to yield a low Coulombic efficiency.^[^ ^48^ ^]^

In stark contrast, capillary cells using 1 m NaPF_6_‐diglyme electrolyte do not produce similar residual SEI structures (with trapped Na particles) that can be observed under our optical microscope (Figure 2h–n, Video S3, Supporting Information). The deposits always appear shiny smooth without forming any disconnected Na particles or flotsams. The corresponding transient voltages were stable.

### 2.3 Chemical Characterizations of the SEI Layers

**Figure 3:**
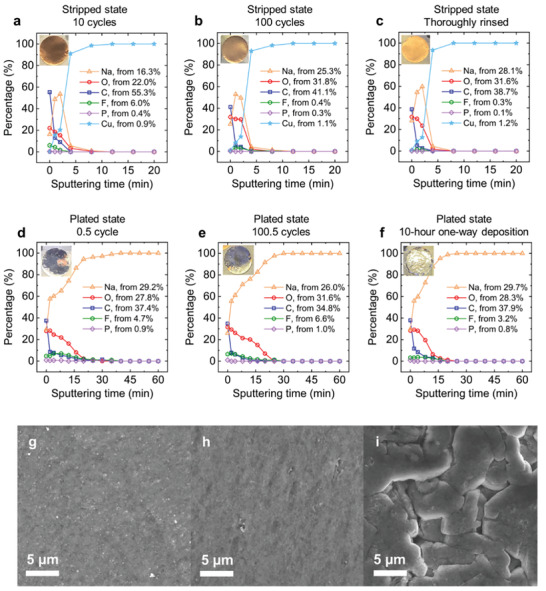
Summary of the XPS elemental concentration profiles along the thickness of various samples. a–c) Stripped‐state samples harvested (a) after 10 full cycles, (b) after 100 full cycles, and (c) after 100 full cycles but rinsed for a longer time. d–f) Plated‐state samples harvested (d) after completing the first half cycle of plating, (e) after the half cycle of plating following 100 full cycles, and (f) after 10 h of one‐way electroplating. Insets are the digital photos of the samples. g,h) SEM images of the (g) sample shown in panel d after the first plating step, and (h) sample shown in panel (f) after 10 h of plating. i) SEM image of the Na deposits obtained in 1 m NaClO_4_‐diglyme electrolyte following the same preparation method as for the sample in panel (d). Experiments of panel a‐f were done in 1 m NaPF_6_‐diglyme electrolyte at 0.5 mA cm^−2^. To estimate the thickness of SEI, a conversion rate^[^ ^3^ ^]^ of 1 nm per minute of sputtering can be adopted.

The results from our operando optical microscopy experiments cannot reveal the details at scales lower than 1 µm, which necessitates the chemical characterization of the surface layers. Cu|Na cells were then fabricated in a practical sandwich structure using stainless steel coin cells. Samples in both the stripped and plated states were harvested and characterized by X‐ray photoelectron spectroscopy (XPS) with depth profiling to identify the electrolyte reduction products, as summarized in **Figure** **3**. The corresponding XPS spectra can be found in Figures S6–S11, Supporting Information, with peak assignments listed in Table S1, Supporting Information.

The Cu|Na cells were cycled at 0.5 mA cm^−2^, with a deposited areal capacity of 1 mAh cm^−2^ in each cycle. The XPS depth profiling shown in Figure 3a reveals the elemental composition of the residual SEI layers left on the Cu current collect after 10 full cycles. Elements from the electrolyte decay to a negligible level in just 4 min of sputtering, which confirms a very thin SEI layer, consistent with the report by Seh et al.^[^ ^3^ ^]^ Surprisingly, however, the SEI layers did not accumulate as the cycle number increases. The Cu current collector harvested from coin cells after 100 full cycles reveal the same thickness of the remaining SEI layers (Figure 3b). This thickness appears also independent from the rinsing time of the sample (Figure 3c). The high Coulombic efficiency indicates that electrical charges used for SEI formation were negligibly low (Figure S12, Supporting Information), supporting the XPS results.

The non‐porous Na deposits, as observed in our capillary cells, allow the XPS characterization of SEI layers on Na deposits, that is, samples in the plated state. It is noteworthy that XPS characterization on deposited Na metal or Li metal was seldom performed before, since the porous nature of the deposited Na or Li metal (i.e., mixtures of metal and SEI) undermines the validity of the depth profiling. Consistent with the observation in the capillary cells (Figure 1), the harvested electrode in the plated state was visually shiny‐smooth. The dark regions in the inset photos of Figure 3d,e are reflections of the stainless‐steel walls of our glove box. See also the confirmation in Video S4, Supporting Information. As shown in Figure 3d–f, regardless of the plating time, that is, the thickness of deposited Na metal, elements from the electrolyte always decay to a negligible level in about 20 min. The sputtering time to reach pure Na in plated samples is notably longer than the time to reach Cu in the stripped samples, but independent from the thickness of the Na deposits. As supported by a control experiment on pristine Na sample (Figure S13, Supporting Information), this longer sputtering time is attributable to the porous oxidized layer formed on the Na deposits during the sample transfer and XPS characterization process. Beneath this top layer, however, the results confirm that the deposited Na metal is indeed nearly 100% pure Na without other elements. As another control experiment demonstrating the impact of the porous structure, the XPS spectra from the plated‐state samples obtained in NaClO_4_‐diglyme electrolyte always show the uniform elemental distribution along the depth, without the Na plasmon peaks even after 45 min of sputtering (Figure S14, Supporting Information). Indeed, the porous Na‐SEI composite structures can accumulate to a considerable thickness as clearly seen in Figure 2a. Scanning electron microscope (SEM) images in Figure 3 clearly reveal that the deposits of Na obtained in NaPF_6_‐diglyme electrolyte are continuous (Figure 3g,h), while those obtained in NaClO_4_‐diglyme electrolyte show clear voids between metal filaments (Figure 3i).

The above results obtained at under‐limiting current densities suggest that the SEI layers on Na in NaPF_6_‐glyme electrolytes do not hinder the formation of shiny‐smooth Na deposits, unlike their Li counterparts that induce root‐growing whiskers.^[^ ^22^, ^48^ ^]^ While the lack of SEI has been confirmed recently on graphite electrode in glyme‐based electrolytes for Na intercalation,^[^ ^42^, ^53^ ^]^ our XPS results do suggest the existence of a nanometer‐thin layer on the Na deposits with typical chemical constituents of SEI.

### 2.4 Cycling Performance of Half and Full Cells

**Figure 4:**
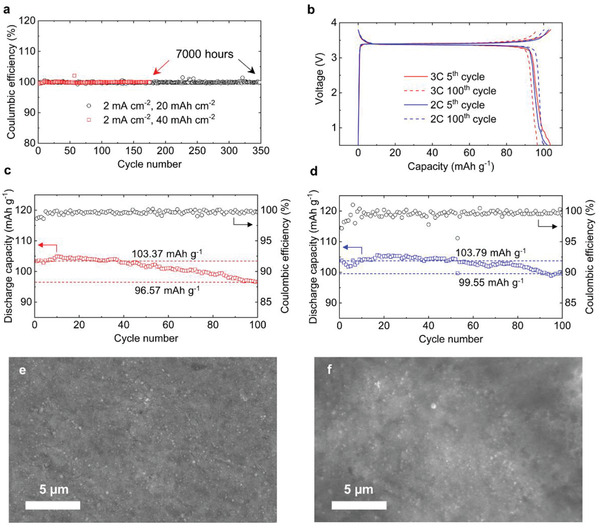
Degradation of half and full cells during galvanostatic cycling. a) Cycling results of Cu|Na half cells at extreme areal capacities. b) Voltage profiles for anode‐free Cu|NVP full cells at 3C (red) and 2C (blue). c,d) Discharge capacity and Coulombic efficiency of the anode‐free Cu|NVP full cell during galvanostatic cycling at 3C rate (c) and 2C rate (d). e,f) SEM images of the Na deposits from the Cu|Na half cell (e) and the anode‐free Cu|NVP full cell (f) after 100 cycles at 3C.

Our discoveries of the shiny‐smooth Na surface and lack of SEI accumulation indicate the possibility of high Coulombic efficiency in practical Na metal cells. Cu|Na half cells were first assembled to test the Coulombic efficiency in galvanostatic cycling experiments with high current densities and areal capacities. At a current density of 2 mA cm^−2^, a near‐100% Coulombic efficiency was obtained for extreme areal capacities of 20 and 40 mAh cm^−2^ (**Figure** **4**a). For the case of 40 mAh cm^−2^, the counter electrode was almost fully depleted. Detailed voltage responses are summarized in Figure S15, Supporting Information. It is worth mentioning that the occasional fluctuations of the Coulombic efficiency are attributable to the dynamic heterogeneity induced at this high current density when the plated Na is being completely stripped from the Cu current collector at the end of each cycle. The near‐unity Coulombic efficiency at these extremely high areal capacities corroborates the ideal reversibility of this system, which agrees with our discovery that only a very thin SEI layer was initially formed and no SEI was accumulated.

Nano‐sized Na_3_V_2_(PO_4_)_3_ (NVP),^[^ ^54^ ^]^ a promising high‐voltage high‐stability Na‐intercalation cathode material,^[^ ^55^ ^]^ was synthesized to fabricate the anode‐free full cells with the bare Cu current collector on the anode side (Cu|NVP). XRD and SEM details of the NVP material are shown in Figure S16, Supporting Information. As demonstrated in Figure 4b, our NVP cathode exhibited a flat voltage plateau at 3.4V, with stable cycling performance (Figure S17, Supporting Information). Our anode‐free Cu|NVP full cell achieved an initial specific capacity of 103.37 mAh g^−1^ during 3C (0.75 mA cm^−2^) charge‐discharge cycling, that is, 87.6% of the theoretical specific capacity of NVP, consistent with the literature.^[^ ^54^ ^]^ After 100 cycles, the cell can still deliver 96.57 mAh g^−1^ specific capacity, which is 93.4% of the initial specific capacity (Figure 4c). Since the Coulombic efficiency can include charge dissipation due to other side reactions,^[^ ^15^, ^31^ ^]^ NIRR and Li inventory retention rate (LIRR) should be used to better capture the degradation process of anode‐free full cells.^[^ ^31^ ^]^ It is not uncommon that when the Coulombic efficiencies are around 99%, the NIRR or LIRR can reach a higher value close to 99.8%.^[^ ^15^, ^56^ ^]^ Using the formula^[^ ^31^ ^]^ 100% × (Q_n_/Q_initial_)^1/n^ = NIRR, the NIRR of our full cell at 3C is 99.93%, higher than published anode‐free Na full cells.^[^ ^14^, ^15^, ^28^, ^29^, ^30^, ^31^ ^]^ Even if we use the maximum capacity of 104.94 mAh g^−1^ at the 10^th^ cycle as the “initial” capacity, the capacity retention after 90 cycles is still 92.0%, yielding a NIRR of 99.91%. The NIRR of our full cell at 2C is even higher, reaching 99.96% per cycle in 100 cycles (Figure 4d). Na deposits in the Cu|Na half cell remain its flatness after 100 cycles (Figure 4e). Those harvested from the Cu|NVP anode‐free full cells with the same cycling condition also appear continuous after 100 cycles (Figure 4f).

The gravimetric energy density of our anode‐free NVP cell at 3C (334.7 Wh kg_NVP_^−1^) is comparable to the theoretical energy densities of successful Li‐ion batteries,^[^ ^57^ ^]^ for example, 385.7 Wh kg_Graphite+LiFePO4_^−1^ from the Graphite|LiFePO_4_ battery. Still, the abundant natural resources of Na and the lack of processing cost on the anode make the anode‐free Na full cells a more sustainable and economical option for energy storage applications.

### 2.5 Impedance Diagnosis of Half and Full Cells

**Figure 5:**
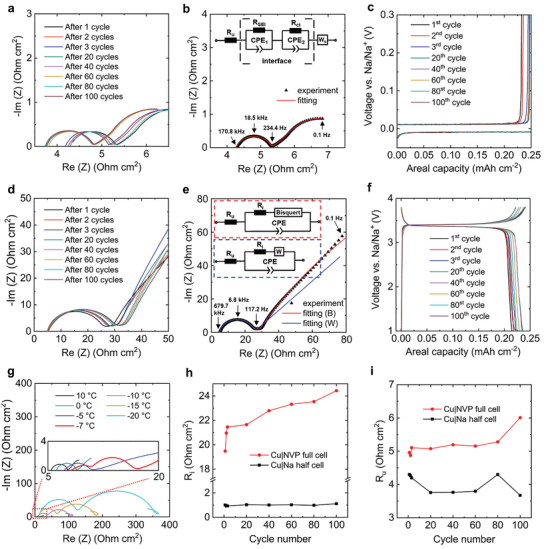
Interfacial stability of half and full cells during cycling. a,d) EIS spectra from 1 to 100 cycles, b,e) typical fitting with the proposed equivalent circuit models, and c,f) the corresponding voltage profiles for Cu|Na half cells (a,b,c) and Cu|NVP full cells (d,e,f). Bisquert is a modified transmission line model for Warburg impedance fitting.^[^ ^65^, ^66^ ^]^ g) Decoupling of the SEI and charge transfer resistances at low temperatures. The freezing point of the 1 m NaPF_6_‐diglyme electrolyte was tested to be −8.2 °C. h) Comparison of the interface resistance. i) Comparison of the uncompensated resistance.

To better understand the degradation and roles of SEI evolution on anode and CEI on the cathode, we further performed EIS tests with both the Cu|Na half cells and the anode‐free Cu|NVP full cells. All cells were cycled at the same current density (3C, 0.75 mA cm^−2^) and same nominal capacities (0.25 mAh cm^−2^). The Nyquist plot of EIS spectra from the half cells after different numbers of cycles (**Figure** **5**a) clearly reveal a nearly perfect semicircle followed by a finite length Warburg behavior,^[^ ^58^ ^]^ consistent with the reported EIS results of Li|Li^[^ ^59^, ^60^ ^]^ and Na|Na symmetric cells.^[^ ^61^ ^]^ While the semicircle is routinely attributed as the charge transfer process, the characteristic frequency at the apex (*ω* = 18.5 kHz) suggests that it is actually from both the SEI and charge transfer processes. As shown in Figure 5b, with the diameter of the semicircle as the resistance across the interface (R = 0.89 Ω, half‐cell electrode area = 1.13 cm^2^), the capacitance responsible for this semicircle can be determined as C = 1/*ω*R = 61 µF. Considering that the charges at the electrolyte|SEI interface and the SEI|electrode interface form a double‐plate capacitor, the dielectric constant for the SEI (ϵ_SEI_) can then be determined by using the general formula C = Aϵϵ_0_/l, where A = 1.13 cm^2^ is the area of the plated Na electrode, l ≈ 4 nm is the estimated thickness of the SEI based on the XPS results using a 1 nm per min conversion rate,^[^ ^3^ ^]^ and *ϵ*_0_ is the permittivity of vacuum. The obtained dielectric constant ϵ = 244 is in the same order of magnitude of the dielectric constants for SEI on Li anode^[^ ^62^ ^]^ and for ion‐conducting polymer electrolytes.^[^ ^63^ ^]^ On the one hand, if we assume that there is no SEI, and Na ions come to the electrode surface for a direct contact before charge transfer, a double‐layer capacitor with a thickness same as the ionic radius of Na (0.102 nm) is formed. Using this new thickness, another dielectric constant is calculated to be 6.2, very close to the reported values (≈7.45) for diglyme.^[^ ^64^ ^]^ Therefore, while one can use just a single R‐C pair to perfectly fit the semicircle, there are actually two interfacial processes as indicated by the equivalent circuit model shown in Figure 5b. The two interfacial capacitors can be deconvoluted at lower temperatures. As can be seen in Figure 5g, the single semicircle clearly deconvolutes into two distinct but connected semicircles when the temperature is lower than −7 °C, consistent with the general expectation that properties of SEI and liquid electrolyte have different dependence on temperature. Nevertheless, the interfacial impedance at room temperature, no matter it is from SEI or charge transfer, remains very low and nearly constant (Figure 5h), which is another compelling confirmation that the initial SEI did not thicken nor accumulate upon cycling. The lack of apparent change of the overpotential in the voltage profiles (Figure 5c) during cycling is yet another piece of evidence that SEI did not thicken or accumulate.

The impedance spectra of full cells show similar behavior (Figure 5d,e) with a nearly perfect semicircle followed by a Warburg tail. The low‐frequency part may be explained by different variants of the Warburg impedance, for example, the Bisquert element derived from a modified transmission line model to account for distributed reactions.^[^ ^65^, ^66^ ^]^ But these choices do not affect the fitting results for the semicircle in the medium‐to‐high frequency range responsible for the interfacial processes. The decrease of the frequency at the apex, from 18.5 kHz for the half cell shown in Figure 5b to 6.8 kHz for the full cell shown in Figure 5e, and the increase of the diameter of the semicircle from 1 Ω cm^2^ to > 19 Ω cm^2^, are clear signs that additional interfacial processes from the cathode are now coupled into the semicircle. As compared in Figure 5h, the interfacial resistance for the full cell steadily grows during cycling, which is attributable to the evolution of the CEI layers. Still, it is noteworthy that the increase of interfacial impedance of the full cell is very slow, and no substantial increase of overpotential was found in the voltage profile (Figure 5f) until toward the end of each discharge.

## 3 Discussion

The lack of repeated formation or accumulation of SEI is supported by two recent theoretical studies. Westman et al.^[^ ^42^ ^]^ performed density functional theory (DFT) calculations and found that the reduction potentials of possible complexes formed in the NaPF_6_‐diglyme electrolyte are all lower than the redox potential of Na/Na^+^, suggesting clearly that this electrolyte is compatible with Na metal. Note that Westman et al.^[^ ^42^ ^]^ investigated the chemical stability by immersing Na metal in the electrolyte for five days. The gas evolution, however, was attributed to the side reaction with PTFE in the pressure cell, rather than the reduction of the solvent. The moisture level in their electrolytes was determined to be lower than 20 ppm. In their latest molecular dynamic simulations, Zhou et al.^[^ ^26^ ^]^ discovered that NaPF_6_‐glyme electrolytes will form an optimal solvation structure, where the PF_6_^−^ anions will be kept far away from the negatively charged electrode surface, minimizing the corrosion effects on Na deposits. Our results that there is no repeated formation of SEI on Na metal during cycling are well supported by these theoretical predictions.

In reality, electrodes do not work in a perfect environment, nor under ideal conditions. The spatiotemporal heterogeneities induced by localized current or electric field may easily drive the system to far‐from‐equilibrium conditions,^[^ ^52^ ^]^ where scattered yet occasional reduction reactions of the electrolyte can still occur. In addition, the trace amount of impurities, for example, water, can affect the SEI formation. In our capillary cell experiments, the amount of water in the electrolyte must be carefully controlled. With a water content higher than 50 ppm, we frequently observed small gas bubbles of pico‐liter size (i.e., diameter of > 20 µm) that interfere with the smooth growths of Na. At a water level of 20–30 ppm, gas bubbles can be avoided, but ingot‐type deposits with shiny‐smooth surfaces cannot be guaranteed. We had to control the water content to be lower than 10 ppm to obtain the shiny‐smooth ingot‐type Na presented here. While a detailed quantitative investigation of the effect of water in various glyme electrolytes is currently underway, the trace amount of water in the range of 10–50 ppm in the LiPF_6_ electrolyte has been confirmed to be able to significantly change the SEI and morphology of Li metal anodes.^[^ ^67^ ^]^

All experiments in this study adopted under‐limiting current densities, at which non‐porous Na ingots were obtained in both the capillary and coin cells. However, dendritic growth could still occur when diffusion limitation is reached. Detailed discussions on diffusion‐limited tip‐growing Na dendrites are out of the scope of our present study, but a typical operando snapshot of electroplating at an over‐limiting current density (Figure S18a, Supporting Information) clearly shows the transition of growth mechanism. It is also important to point out that, when relatively high current densities are applied, the macroscopic heterogeneous separator|electrode interface, not the homogeneous electrolyte|electrode interface, would naturally select a few penetration channels (black spots in Figure S18b, Supporting Information), through which the ionic flux must have been strongly localized to enable the metal penetration. Within these isolated channels, over‐limiting current densities and diffusion‐limited tip‐growing dendrites must have been triggered.^[^ ^52^ ^]^

**Figure 6:**
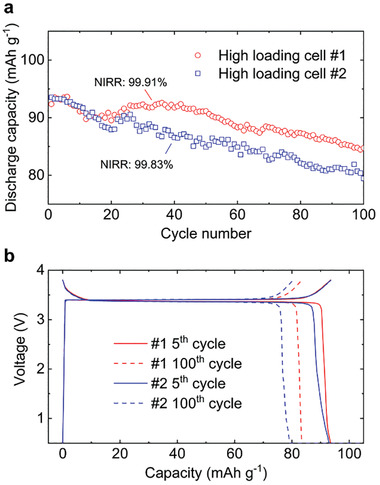
Degradation of high‐loading anode‐free full cells during galvanostatic cycling. a) Cycling performance and b) selected voltage profiles of the high‐loading anode‐free Cu|NVP full cells. The NVP loading is around 6.2 mg cm^−2^ for both cells, yielding a similar initial specific capacity of around 93 mAh g^−1^ at 2C rate (1 mA cm^−2^), and the similarly high NIRRs of 99.91 and 99.83%.

Our results demonstrated a successful comprehensive solution to both the non‐uniform growths and the low Coulombic efficiency of Na metal anodes.^[^ ^2^, ^9^ ^]^ Compared with the latest reports using diglyme electrolytes for Na metal battery,^[^ ^25^, ^26^, ^27^ ^]^ we did not assemble excess Na in the coin cell for full cell cycling tests. Compared with the anode‐free Na metal batteries by Cohn et al.,^[^ ^14^, ^15^ ^]^ we did not engineer an additional nano‐carbon nucleation layer, but only used the simplest bare copper foil. The near‐unity Coulombic efficiency achieved in Na metal half cells working at extreme areal capacities, as well as the impedance diagnosis upon cycling, suggest that the performance degradation of our anode‐free full cells was dominated by the cathode. Our NVP cathode exhibits excellent stability in the Na|NVP half cells (Figure S17, Supporting Information). The degradation becomes more obvious in the anode‐free Cu|NVP full cells, in which the Na loss to CEI can no longer be compensated by excess Na. To fully exploit the ideal stability of the metal anode, high‐loading Na‐ion cathodes must be developed. **Figure** **6** demonstrates the excellent cycling efficiency of anode‐free full cells with thicker NVP electrodes (≈ 6.2 mg cm^−2^). However, preparing ultra‐thick electrodes with a loading > 10 mg cm^−2^ without cracking,^[^ ^68^ ^]^ yet with satisfactory tortuosity,^[^ ^69^ ^]^ is challenging.^[^ ^14^, ^15^ ^]^ Fine‐tuning the chemistry and viscosity of the solvents^[^ ^68^ ^]^ for making the slurry and controlling the drying process may help realize high‐capacity high‐power anode‐free Na batteries that can outperform Li‐ion batteries.

## 4 Conclusion

With our special glass capillary cells, we have demonstrated for the first time the self‐modulated shiny‐smooth non‐porous growth of an alkali metal ingot in liquid electrolytes, which can be cycled reversibly without producing Na whiskers, mosses, gas bubbles, or disconnected Na particles. Contradictory to the prevailing understandings on SEIs, we discovered that there was no repeated SEI formation during cycling, despite the significant volume change of the metal anode during cycling. The very initial SEI components that can be detected by XPS depth profiling do not thicken or accumulate regardless of the deposition thickness or cycling history of the Na electrodes. Our discovery is very well supported by recent theoretical studies that the NaPF_6_‐diglyme electrolyte is thermodynamically stable against Na metal. The simplest anode‐free Na full cells in the configuration of Cu|Na_3_V_2_(PO_4_)_3_, without excess Na metal or special modifications of the bare copper current collector, exhibit stable cycling performance. A NIRR of 99.93% per cycle during galvanostatic cycling at 3C (0.75 mA cm^−2^) has been achieved. Our work proved the possibility and significance to achieve the ideal interfacial stability of an alkali metal in liquid electrolytes, for which our operando microcapillary cell experiment provides the necessary and effective method to identify the unexpected performance‐limiting dynamic subtleties that hard to be detected by other methods. Based on the insights, future research on the solvation structures and interfacial reaction kinetics may eventually enable similar shiny‐smooth non‐porous growths of other metal anodes in liquid electrolytes.

## 5 Experimental Section

##### Materials

1,2‐Dimethoxyethane (monoglyme, anhydrous, 99.5%), diethylene glycol dimethyl ether (diglyme, anhydrous, 99.5%), triethylene glycol dimethyl ether (triglyme, 99%), tetraethylene glycol dimethyl ether (tetraglyme, ≥99%), ethylene carbonate (EC, anhydrous, 99%), dimethyl carbonate (DMC, anhydrous, ≥99%), sodium cubes (99.9%), vanadium oxide (V_2_O_5_, ≥99.6%), ammonium phosphate monobasic (NH_4_H_2_PO_4_, anhydrous, ≥98%), sodium carbonate (Na_2_CO_3_, anhydrous, ≥99.5%), L‐ascorbic acid (anhydrous, ≥99%), poly(ethylene glycol) (PEO, M_n_ 400), and N‐Methyl‐2‐pyrrolidinone (NMP, ≥99%) were purchased from Sigma‐Aldrich. All the solvents were dried using molecular sieves for at least 3 days before use. Sodium hexafluorophosphate (NaPF_6_, >99%, Alfa Aesar) and sodium perchlorate (NaClO_4_, anhydrous, ACS reagent, Acros Organics) were purchased from Fisher Scientific. Salts were dried at 100 °C for 48 h before use. Water content was measured by a coulometric Karl Fischer titrator (Metrohm). For the NaPF_6_‐based electrolytes (with DME, diglyme, tetraglyme, or EC/DMC), the moisture level was in the range of 5–10 ppm. For the NaClO_4_‐based electrolytes (with DME, diglyme, triglyme, tetraglyme, or EC/DMC) the moisture level was in the range of 28–35 ppm. 0.01‐inch diameter stainless steel wires and 0.03‐inch thickness polyvinylidene fluoride (PVDF) sheets were purchased from McMaster‐Carr. The G‐1 glass capillaries were purchased from Narishige Co., Ltd. Polypropylene‐Polyethylene‐Polypropylene (PP‐PE‐PP) tri‐layer battery separator (Celgard 2325, 25 µm thickness), copper foil for battery anode substrate (9 µm thickness), aluminum foil for battery cathode substrate (15 µm thickness), conductive acetylene black, and HSV900 PVDF binder were purchased from MTI Corporation.

##### Synthesis

The synthesis process of nano‐sized Na_3_V_2_(PO_4_)_3_ (NVP) followed the reported hydrothermal assisted sol‐gel method.^[^ ^54^ ^]^ V_2_O_5_, NH_4_H_2_PO_4_, Na_2_CO_3_, L‐ascorbic acid, and PEO 400 were mixed with the desired molar ratio in distilled water. The mixture was kept at 180 °C in an autoclave for 40 h, followed by open heating at 95 °C to evaporate the water. The obtained precursor was preheated at 350 °C for 4 h and then calcinated at 750 °C for 6 h in the flowing Ar atmosphere to obtain the final product.

##### Electrode Preparation

Nano‐sized NVP, conductive acetylene black, and PVDF binder were thoroughly mixed into slurry with NMP at a weight ratio of 8:1:1. The slurry was cast onto the aluminum foil and dried at 120 °C overnight before cutting into disk electrodes. The diameter of the disk electrode was 8 mm, that is, a geometric area of 0.5 cm^2^. The mass loading of NVP was around 2.3 mg cm^−2^ for the tests shown in Figures 4 and 5, and around 6.2 mg cm^−2^ for the high‐loading tests shown in Figure 6. The diameter of the Cu current collector was 12 mm. In Cu|Na half cells, diameters of the Na electrode and the Cu current collector were 12 mm, that is, a geometric area of 1.13 cm^2^.

##### Cells Fabrication and Electrochemical Testing

All cells were assembled in an Ar‐filled glove box with H_2_O and O_2_ concentration < 0.5 ppm. The glass capillaries were pulled 7 mm longer with a vertical type micropipette puller (PC‐10, Narishige Co., Ltd). The pulled capillary was fixed onto a piece of glass slide using epoxy. Electrolytes were filled in the capillary by the capillary effect from one side. Two pieces of sodium were then pushed by stainless steel wires to form a Na|Na symmetric cell. The separation between two electrodes varies from ≈380 to ≈3500 µm depending on the aims of the experiments. In situ images were captured by an optical microscope (MU500, AmScope). 2025‐type coin cells were fabricated for preparing the samples for XPS characterization and for long‐term cycling tests. The Cu|Na half cells were assembled with the configuration of Cu|separator|Na, where one layer of the PP‐PE‐PP tri‐layer separator was used. The Cu|NVP anode‐free full cells with the configuration of Cu|separator|NVP were also constructed in 2025‐type coin cells. Electrochemical tests were conducted with a Gamry potentiostat (Reference 600+, Gamry Instruments), an Arbin battery tester (LBT 20 084, Arbin Instruments), and a Land battery testing system (CT3001A, Lanhe instruments). The impedance tests were performed at deposited states with a frequency range from 5 MHz to 0.1 Hz.

##### Characterization

For XPS characterization, Cu|Na sandwich cells were disassembled after cycling at 0.5 mA cm^−2^ for 1 mAh cm^−2^ (without considering the porosity) for desired cycle numbers to examine the SEI at deposited or stripped states. Samples were washed in diglyme before fixing onto the XPS holder. The standard washing procedure was to soak the sample in 10 mL of diglyme solvent for 30 s with gentle shaking. For the “thoroughly rinsed” sample, in addition to the standard procedure, the sample was laid flat and washed by dispensing about 1 mL diglyme from a plastic dropper. The XPS holder was transferred into the chamber within an argon‐filled XPS transfer kit. XPS characterization was performed using Physical Electronics 5000 VersaProbe II Scanning ESCA Microprobe. The depth profiling was carried out by the argon‐ion sputtering at 2 kV and 1 µA at a 2 × 2 mm area. For XRD characterization, Bruker D8 advance X‐ray diffractometer was utilized. Si crystal zero diffraction plate purchased from MTI Corporation was used as a powder sample holder. For SEM imaging, Thermofisher Quattro S environmental SEM was utilized.

## Conflict of Interest

The authors declare no conflict of interest.

## Author Contributions

P.B. conceived and supervised the study. B.M. and P.B. designed the experiments. B.M. performed the experiments and carried out the analyses. Y.L. helped with the SEM, XPS and XRD experiments and analyses. B.M and P.B. wrote the manuscript. All authors discussed the results and revised the manuscript.

## Supporting information

Supporting Information

Supporting Video 1

Supporting Video 2

Supporting Video 3

Supporting Video 4

## Data Availability

The data that support the findings of this study are available from the corresponding author upon reasonable request.
