# Supplementary material for: Dynamic Interfacial Stability Confirmed by Microscopic Optical Operando Experiments Enables High‐Retention‐Rate Anode‐Free Na Metal Full Cells
Source: Adv Sci (Weinh). 2021 May 3;8(12):2005006. doi: 10.1002/advs.202005006 (PMC8224441; doi:10.1002/advs.202005006)
Supplement: Supplementary file 1 — Supporting Information [file ADVS-8-2005006-s004.pdf]

© 2021 Wiley-VCH GmbH

ADVANCED SCIENCE
Open Access

Supporting Information

for *Adv. Sci.*, DOI: 10.1002/advs.202005006

Dynamic Interfacial Stability Confirmed by Microscopic Optical *Operando* Experiments Enables High-retention-rate Anode-free Na Metal Full Cells

*Bingyuan Ma, Youngju Lee, and Peng Bai**

Supporting Information

Dynamic Interfacial Stabilities Revealed by Microscopic Optical *Operando* Experiments Enables High-retention-rate Anode-free Na Metal Full Cells

Bingyuan Ma[1], *Youngju Lee*[1], *Peng Bai*[1,2,*]

[1] Department of Energy, Environmental & Chemical Engineering, Washington University in St. Louis, St. Louis, MO 63130, United States of America

[2] Institute of Materials Science and Engineering, Washington University in St. Louis, St. Louis, MO 63130, United States of America

*Correspondence to: pbai@wustl.edu

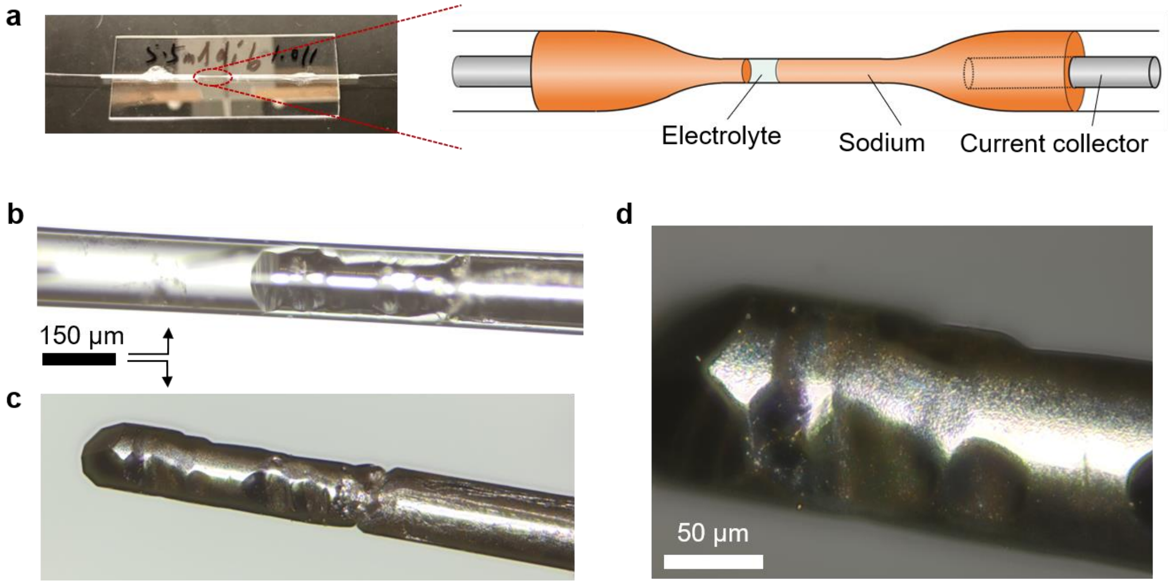

Figure S1. a) The structure of the capillary cells. b,c) The deposition and the pristine sodium b) before and c) after removed from the capillary cell. d) The higher magnification image of the sodium deposition.

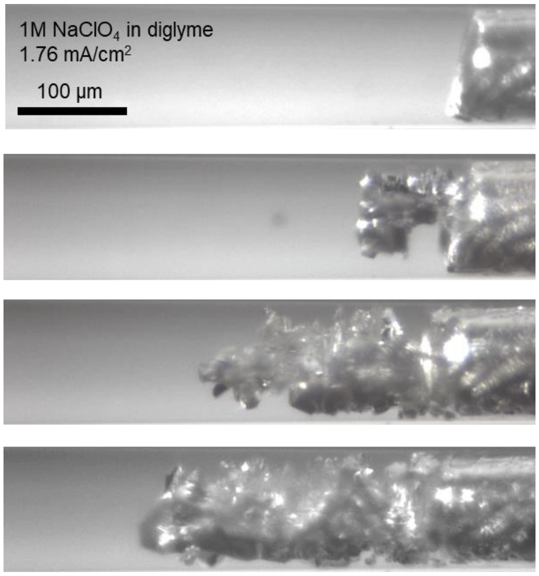

Figure S2. Snapshots of the sodium metal deposition at an under-limiting current in 1 M $NaClO_4$-diglyme electrolyte.

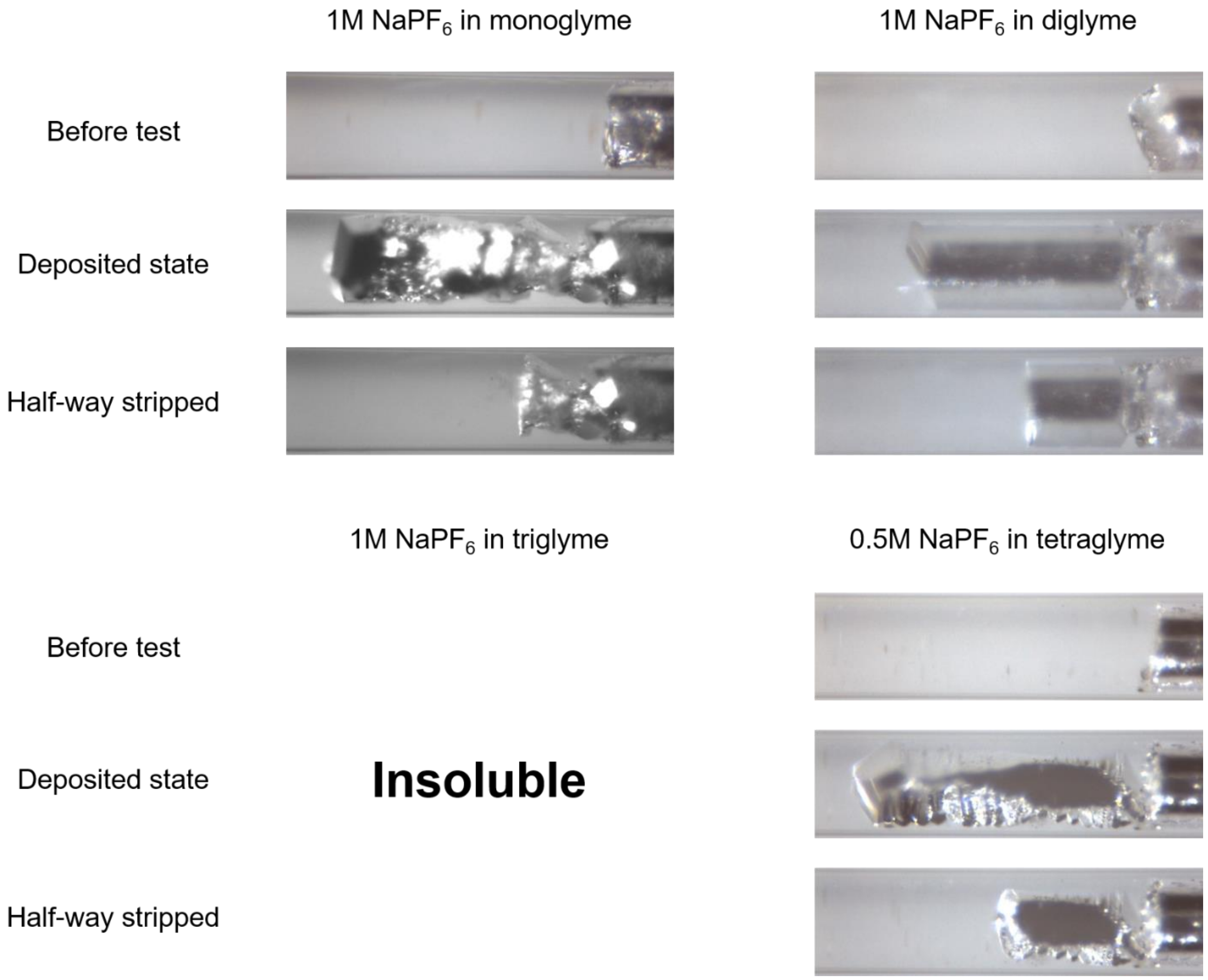

Figure S3. Album of Na deposition and dissolution in $NaPF_6$-glyme systems. Experiments were done at 2 mA cm^{-2}.

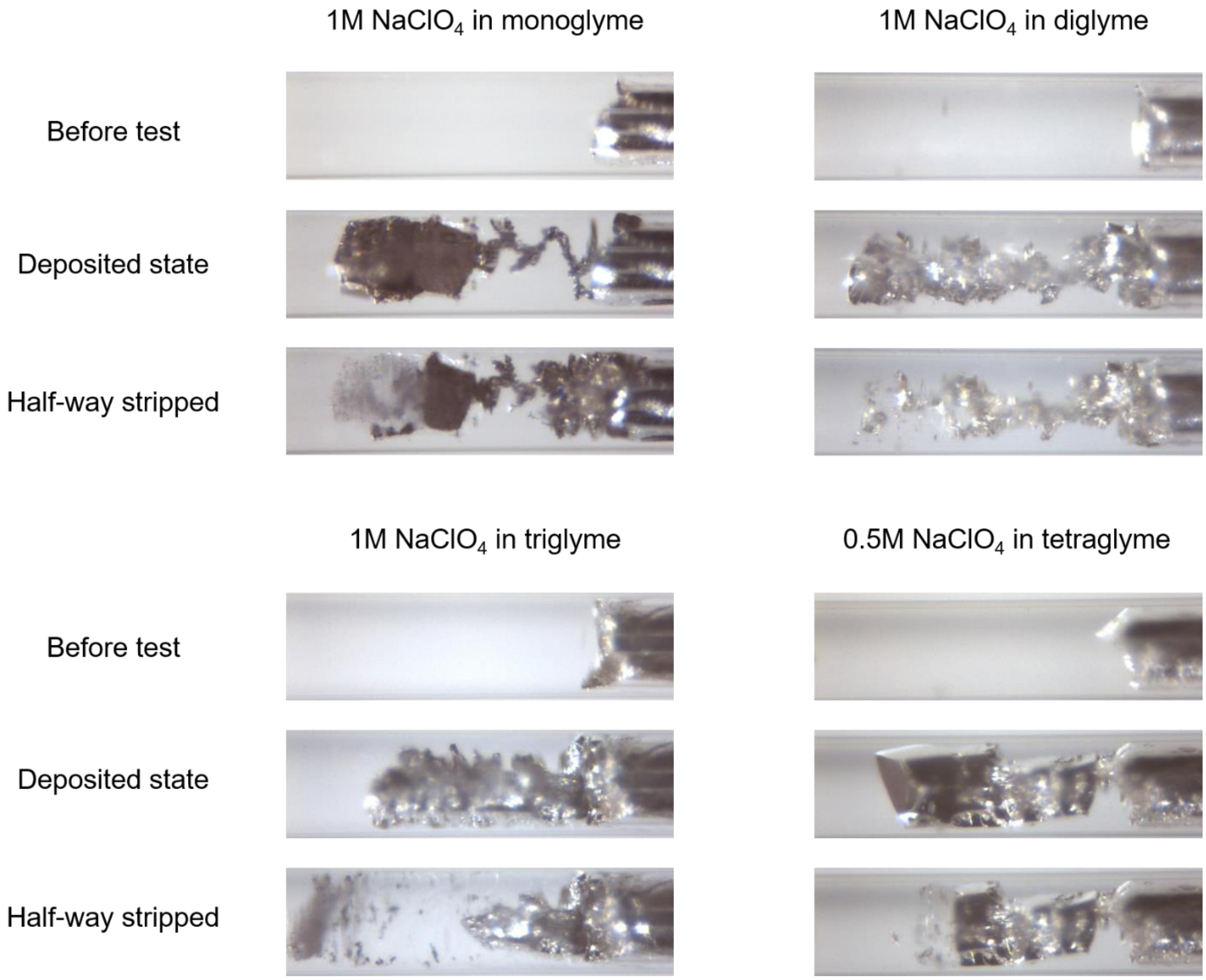

Figure S4. Album of Na deposition and dissolution in $NaClO_4$-glyme systems. Experiments were done at 2 mA cm^{-2}.

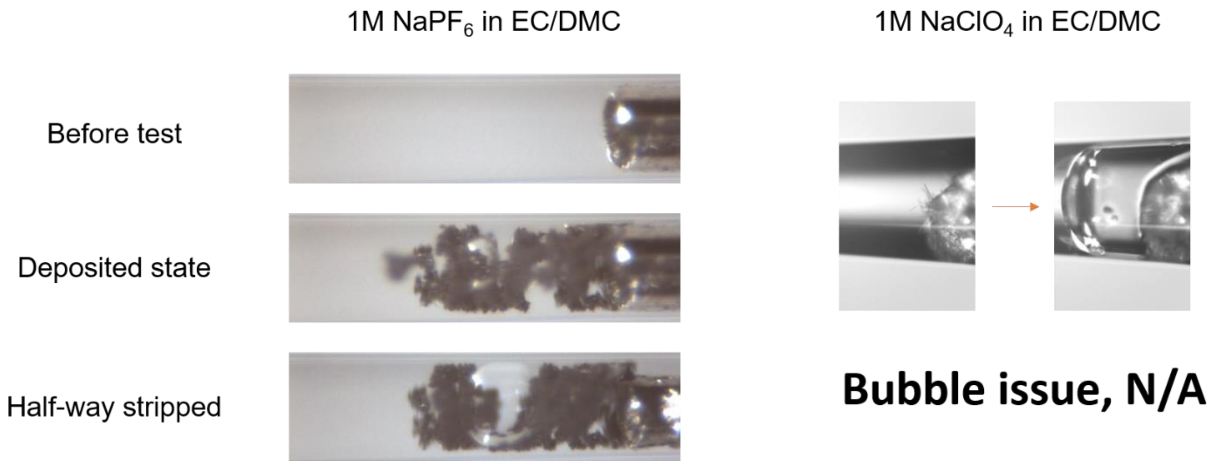

Figure S5. Album of Na deposition and dissolution in carbonate-based systems. Experiments were done at 2 mA cm^{-2}.

Table S1. Binding energy and the peak assignments of the SEI component

Element	Binding energy (eV)	Peak assignment	Species	Reference
C 1s	284.8	C-C, C-H	RH_2ONa	[1–3]
	286.5	C-O	RH_2ONa	[1–3]
	288.9	C=O	Na_2CO_3	[1,2,4–6]
O 1s	529.3	Na-O	Na_2O	[1,7,8]
	530.9	C=O	Na_2CO_3	[1,2,4]
	532.5	C-O	RH_2ONa	[1–4]
	533-537	Na KLL		[1,4,7]
F 1s	683.9	Na-F	NaF	[1,3]
	687.3	P-F	Na_xPF_y, $Na_xPO_yF_z$	[1,3]
Na 1s	1071.8	Na-O, Na-F	Na_2O, NaF,	[1,4,7]
	1070.1	Na-Na	Na metal	[5]
P 2p	136.7	P-F	Na_xPF_y, $Na_xPO_yF_z$	[1,3]
Cu 2p3/2	932.9	Cu	Cu	[1,3]

To the best of our knowledge, the above assignments represent the prevailing choices with confirmations in the literature. While peak assignments for samples in the stripped state (without metallic Na) are relatively straightforward to make, interpretations of XPS data for samples in the plated state (with Na metal) are intrinsically difficult, due to not only the overlapping between the O spectra with the Na KLL peaks[1], but also the fast reaction between Na and the possible trace amount of O_2 during the transfer and characterization processes, even though the sample was transferred in a standard air-tight holder and a high vacuum was always maintained during the XPS characterization.

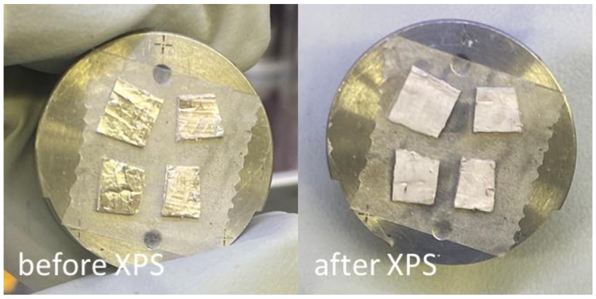

For our O 1s spectra, the peak at 529.3 eV is consistent with the Na-O peak[6] from Na_2O. The peak that has the strongest intensity at 530.9 eV was assigned to C=O[1,2,4], instead of Na-O,[3] indicating the outer Na_2O layer further reacted with moisture and CO_2. The small peak at 532.5 eV is therefore assigned to C-O.[1–4] The depth profiles of C-O and C=O from O 1s spectra are consistent with those from the C 1s spectra. Peaks located higher than 533 eV were assigned as Na KLL peaks[1], rather than C-O[3], as the binding energies for metal oxides were suggested to be lower than this value[1].

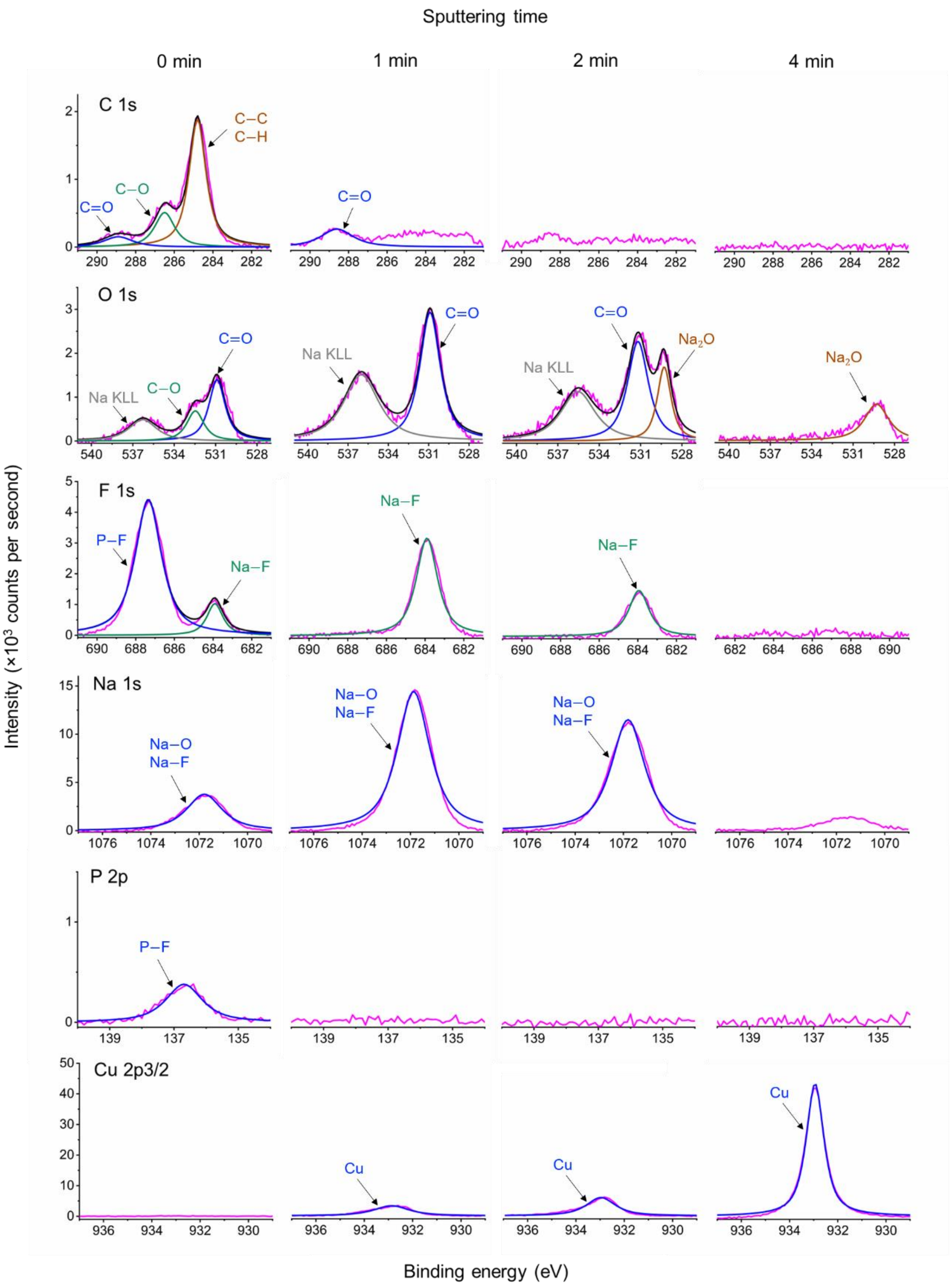

Figure S6. Details for Figure 3a. XPS characterization of the SEI at stripped state after 10 full cycles in 1 M $NaPF_6$-diglyme electrolyte.

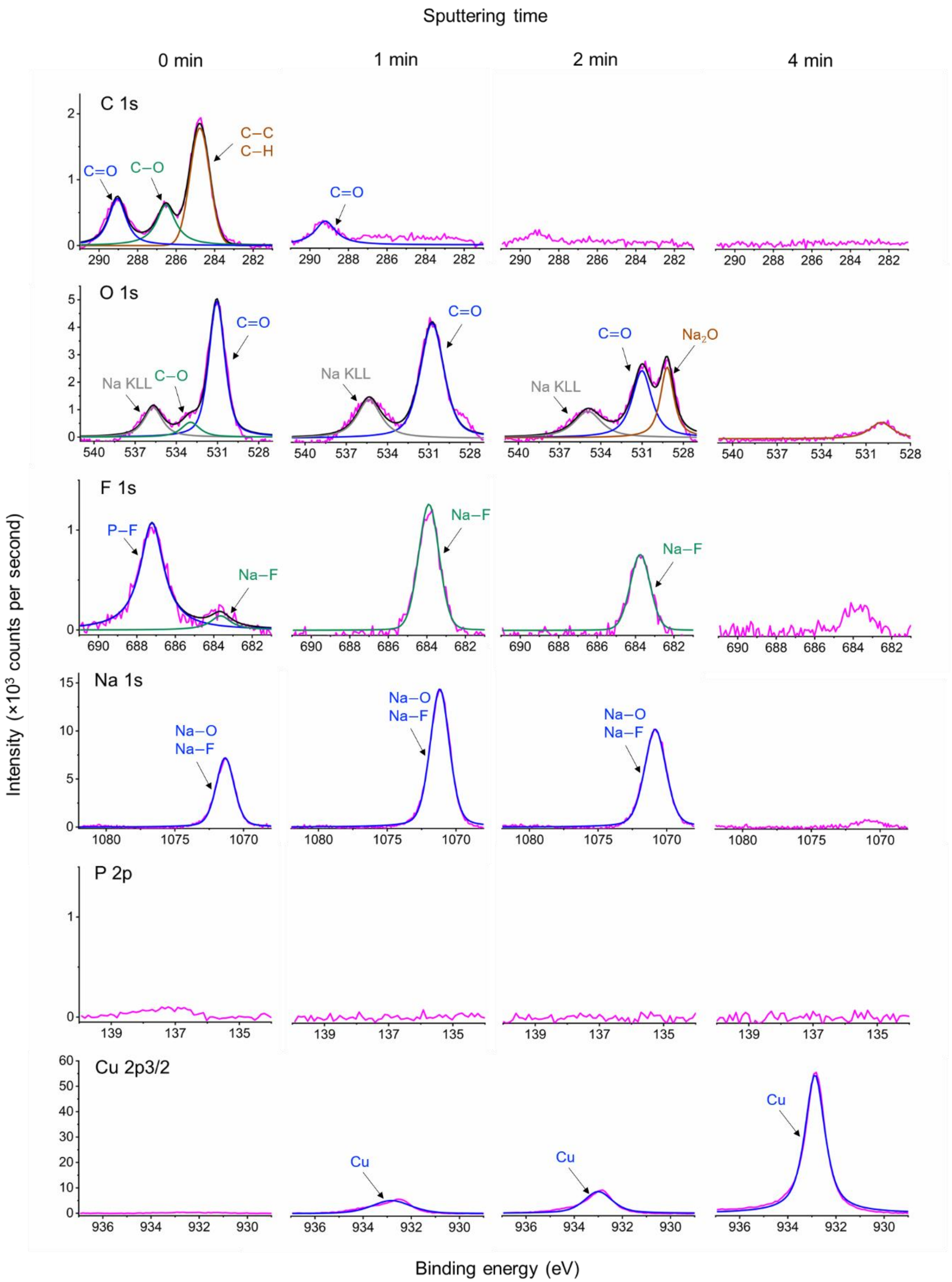

Figure S7. Details for Figure 3b. XPS characterization of the SEI at stripped state after 100 full cycles in 1 M $NaPF_6$-diglyme electrolyte.

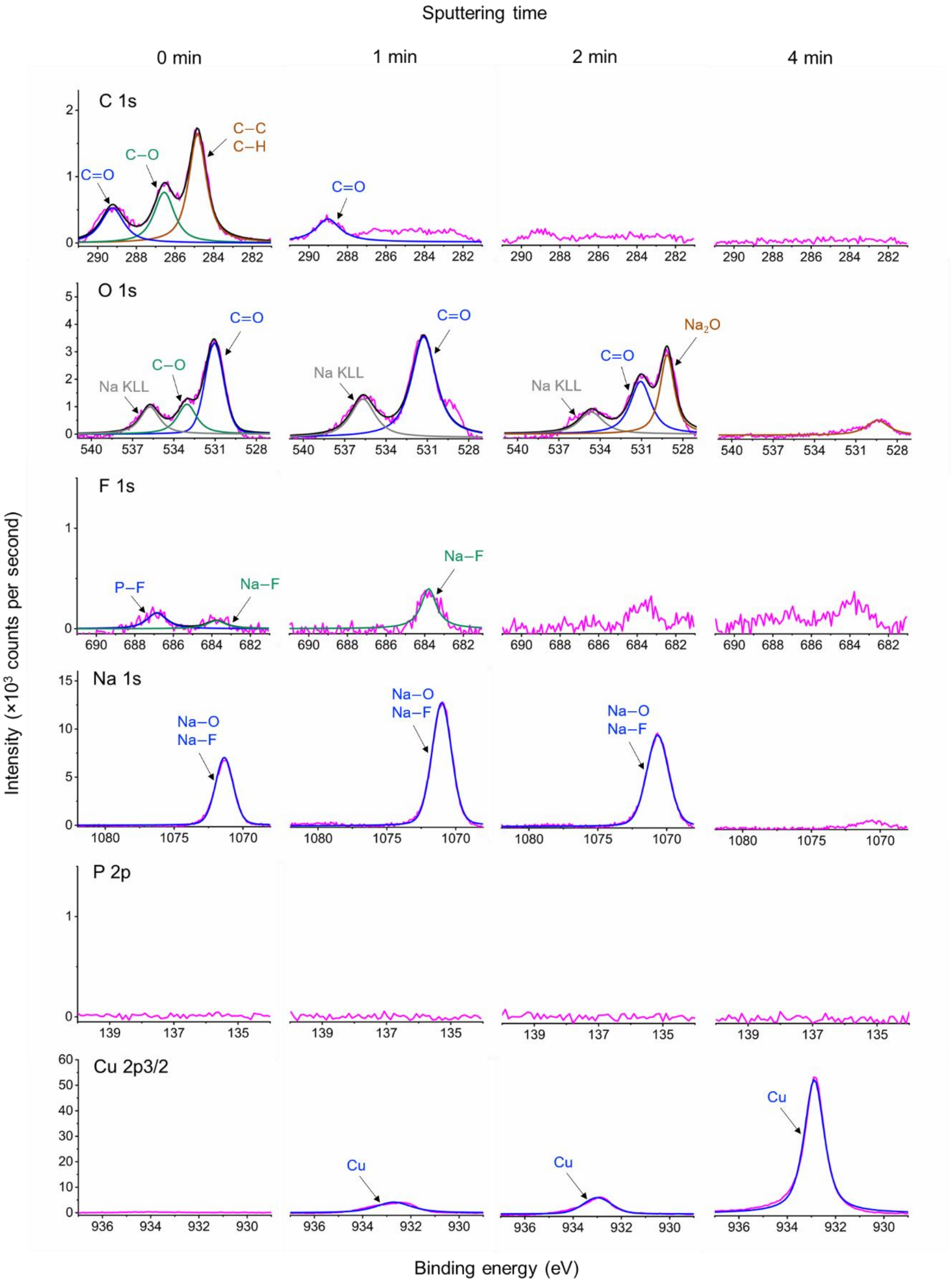

Figure S8. Details for Figure 3c. XPS characterization of the SEI at stripped state after 100 full cycles and rinsed for longer time in 1 M $NaPF_6$-diglyme electrolyte.

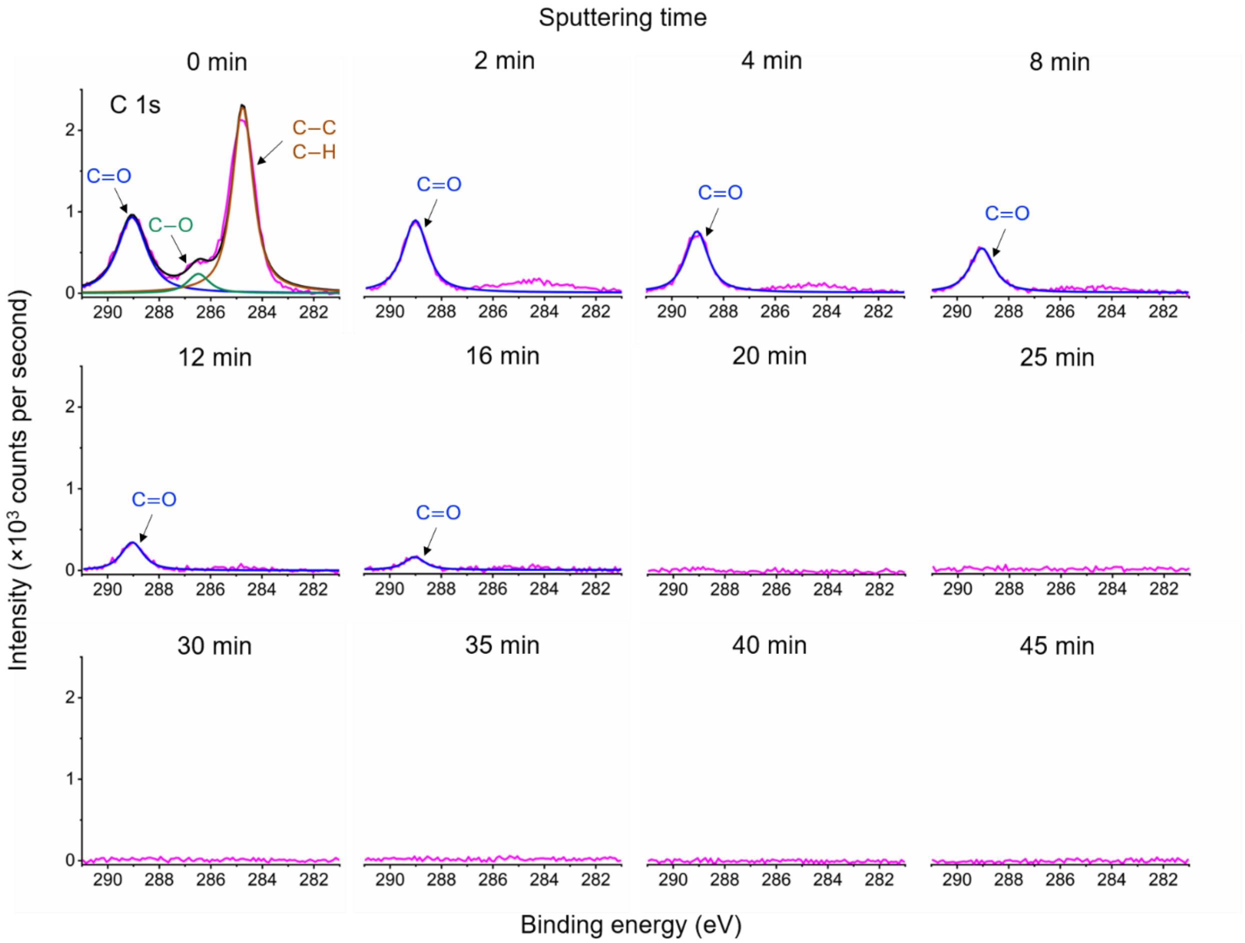

Figure S9a. Details for Figure 3d. XPS characterization of the SEI at deposited state after completing the first half cycle of deposition in 1 M $NaPF_6$-diglyme electrolyte. This figure shows the C 1s spectra.

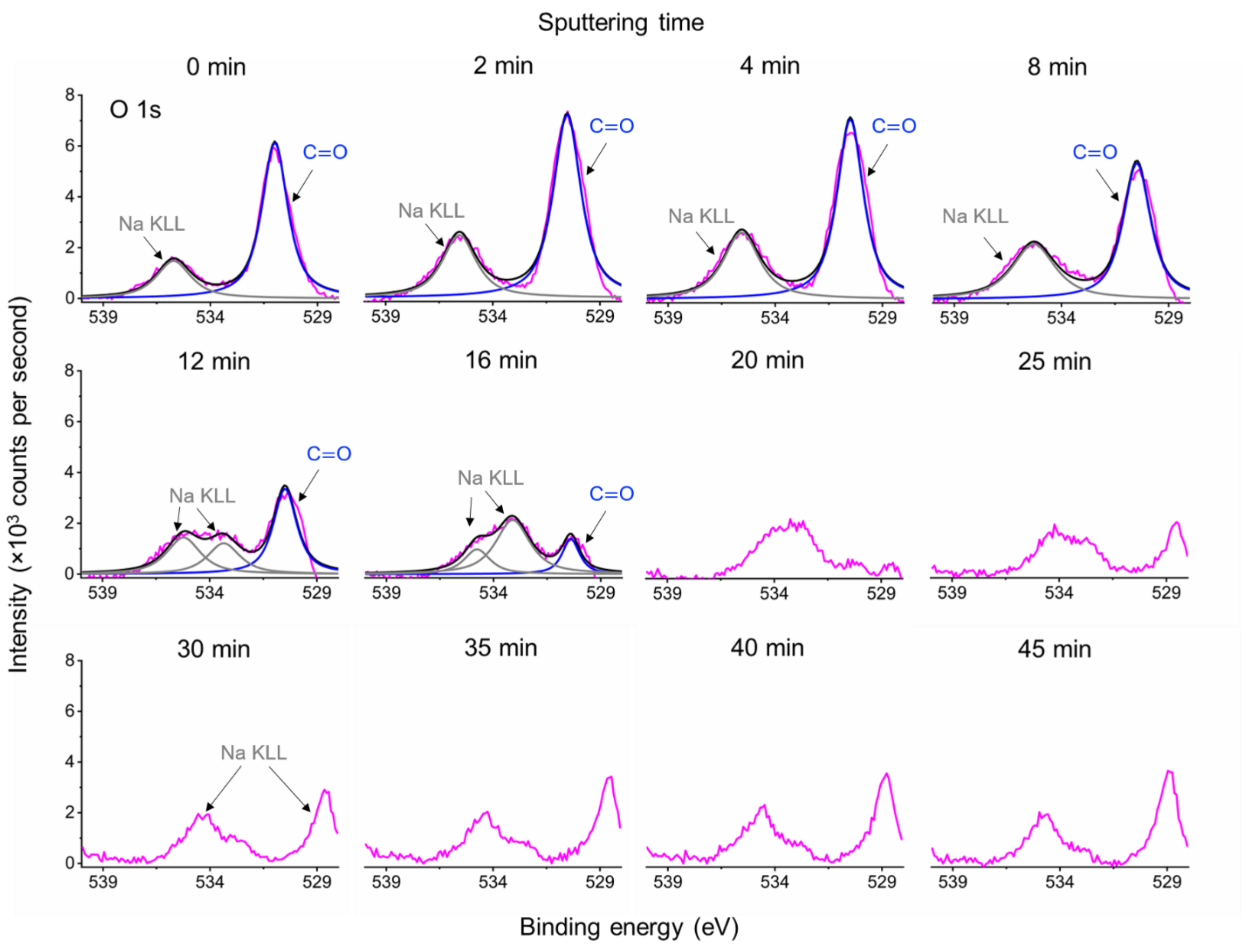

Figure S9b. Details for Figure 3d. XPS characterization of the SEI at deposited state after completing the first half cycle of deposition in 1 M $NaPF_6$-diglyme electrolyte. This figure shows the O 1s spectra.

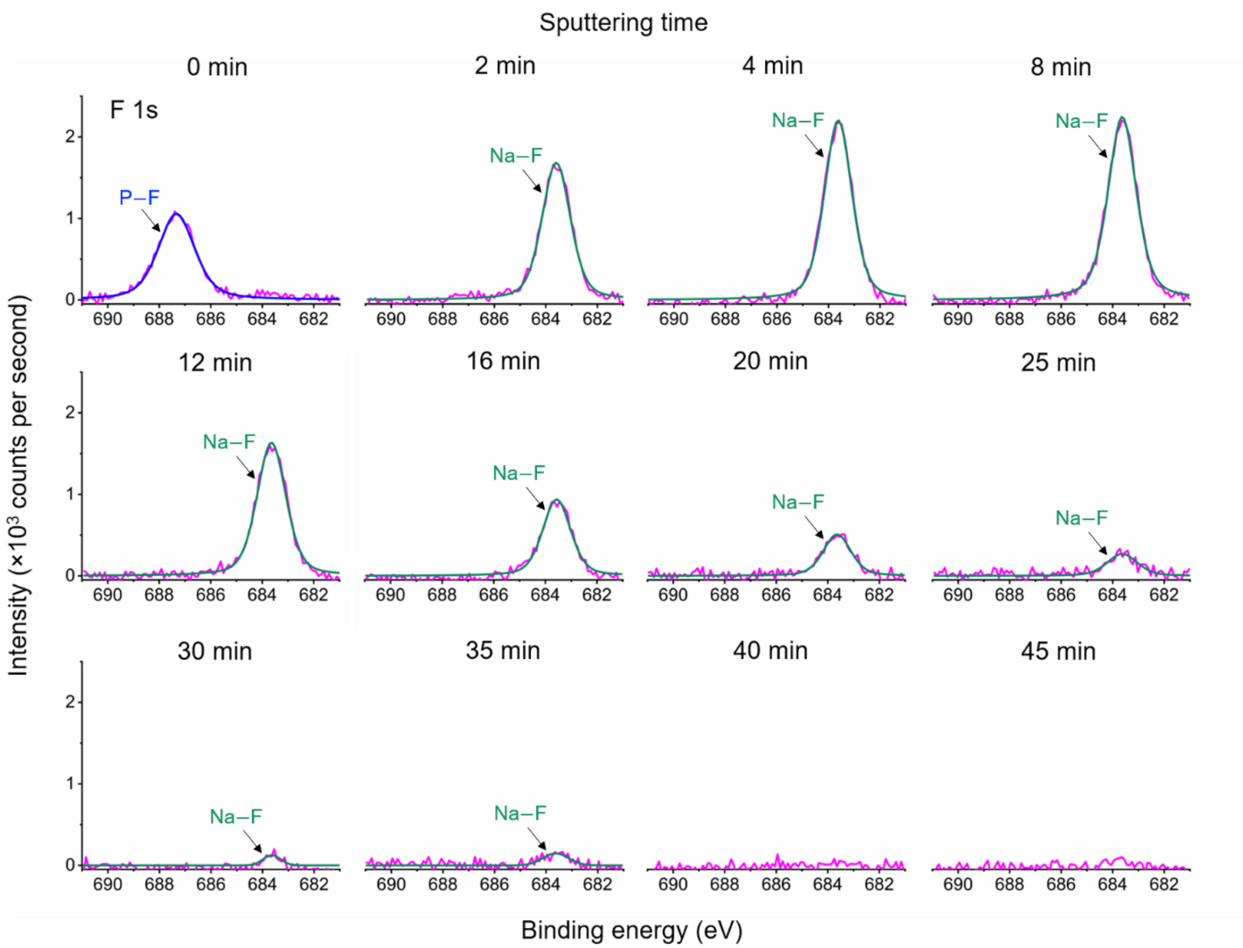

Figure S9c. Details for Figure 3d. XPS characterization of the SEI at deposited state after completing the first half cycle of deposition in 1 M $NaPF_6$-diglyme electrolyte. This figure shows the F 1s spectra.

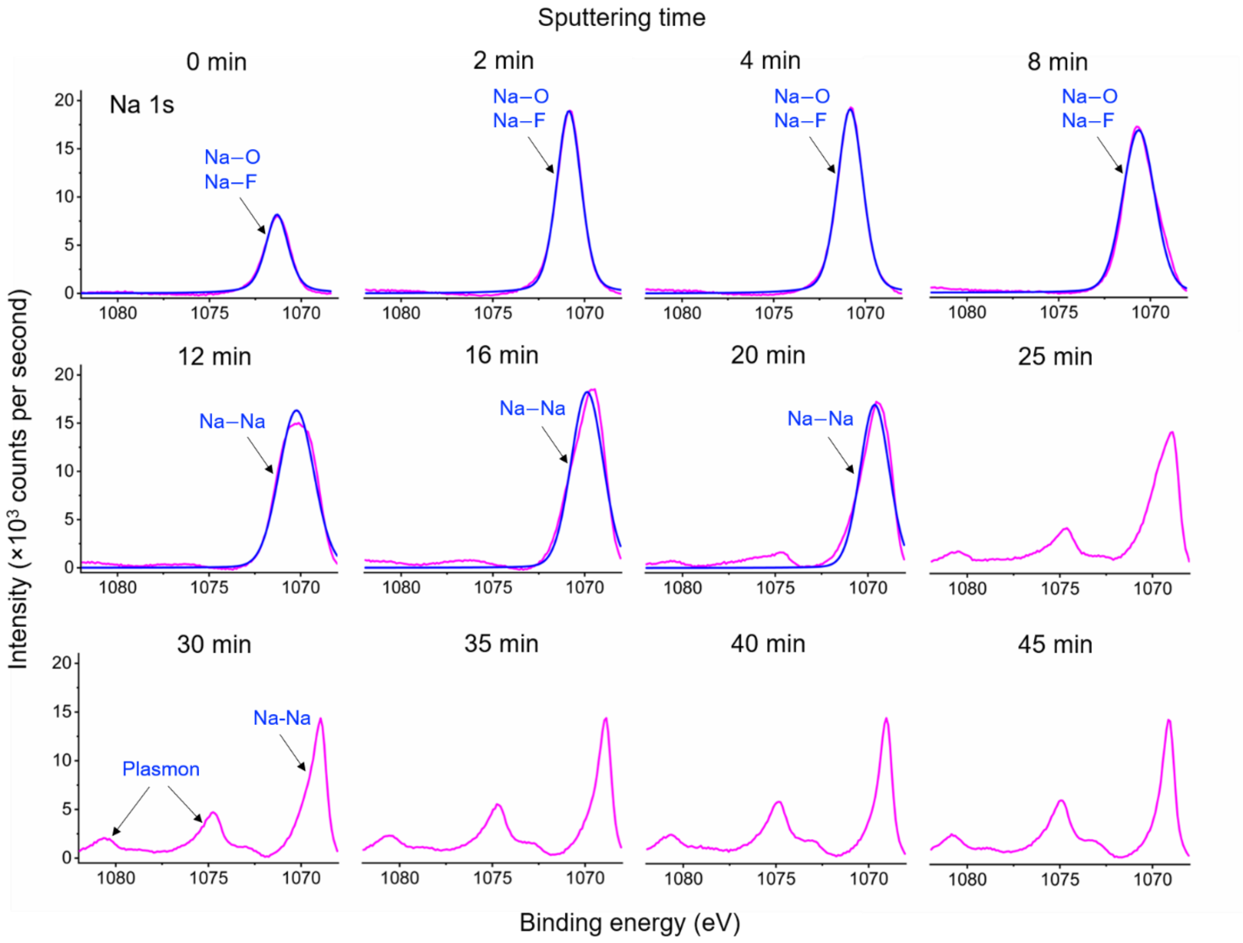

Figure S9d. Details for Figure 3d. XPS characterization of the SEI at deposited state after completing the first half cycle of deposition in 1 M $NaPF_6$-diglyme electrolyte. This figure shows the Na 1s spectra.

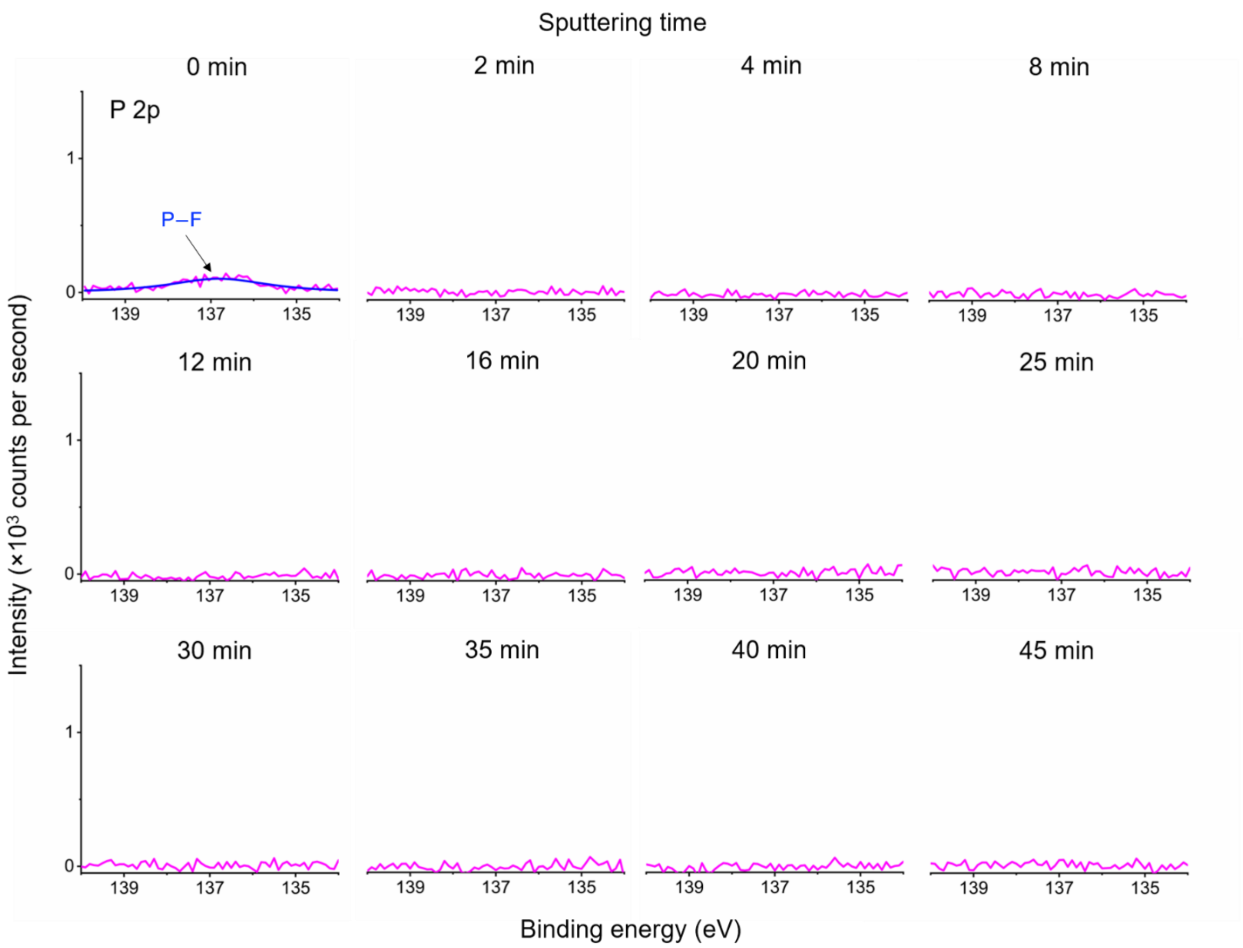

Figure S9e. Details for Figure 3d. XPS characterization of the SEI at deposited state after completing the first half cycle of deposition in 1 M $NaPF_6$-diglyme electrolyte. This figure shows the P 2p spectra.

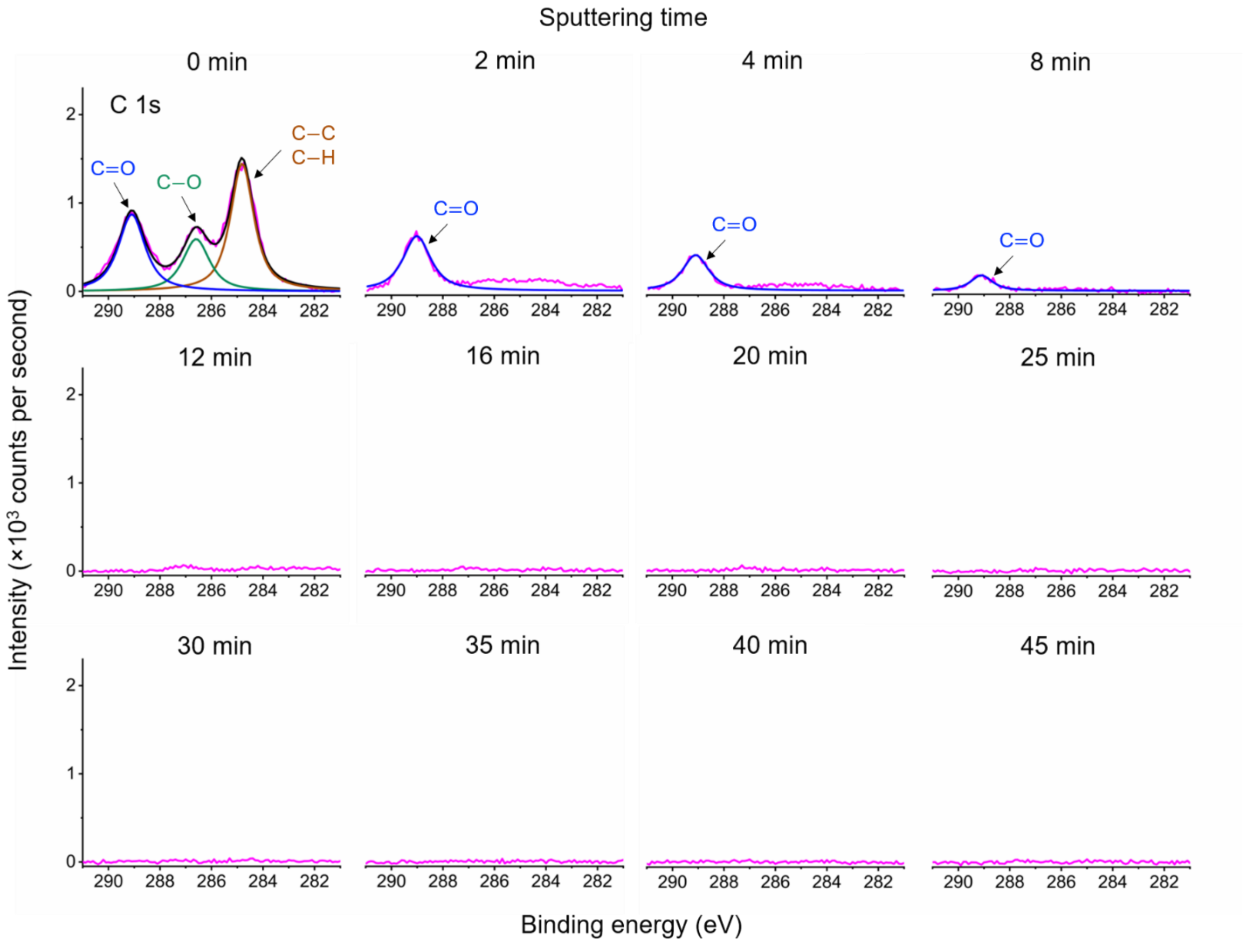

Figure S10a. Details for Figure 3e. XPS characterization of the SEI at deposited state after 100.5 cycles in 1 M $NaPF_6$-diglyme electrolyte. This figure shows the C 1s spectra.

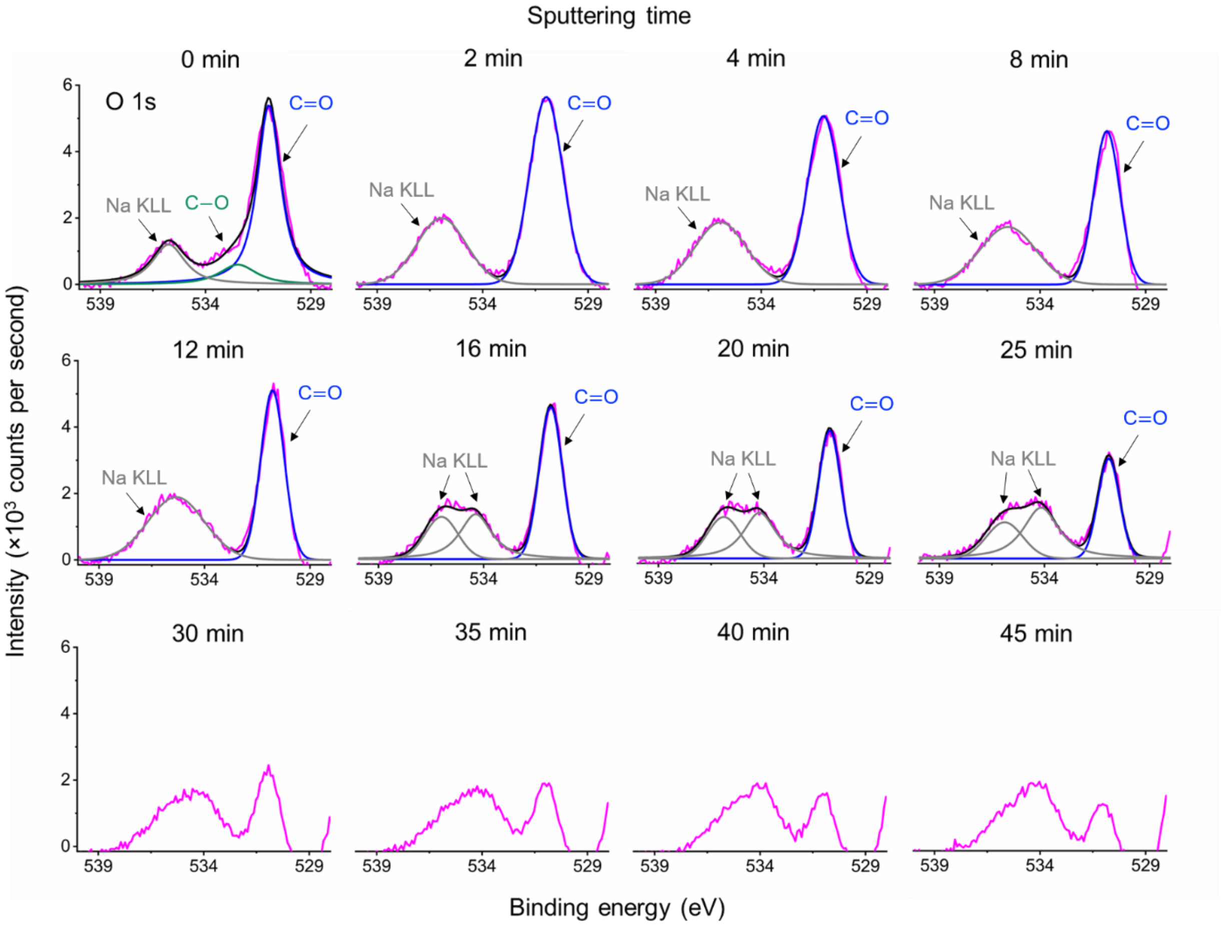

Figure S10b. Details for Figure 3e. XPS characterization of the SEI at deposited state after 100.5 cycles in 1 M $NaPF_6$-diglyme electrolyte. This figure shows the O 1s spectra.

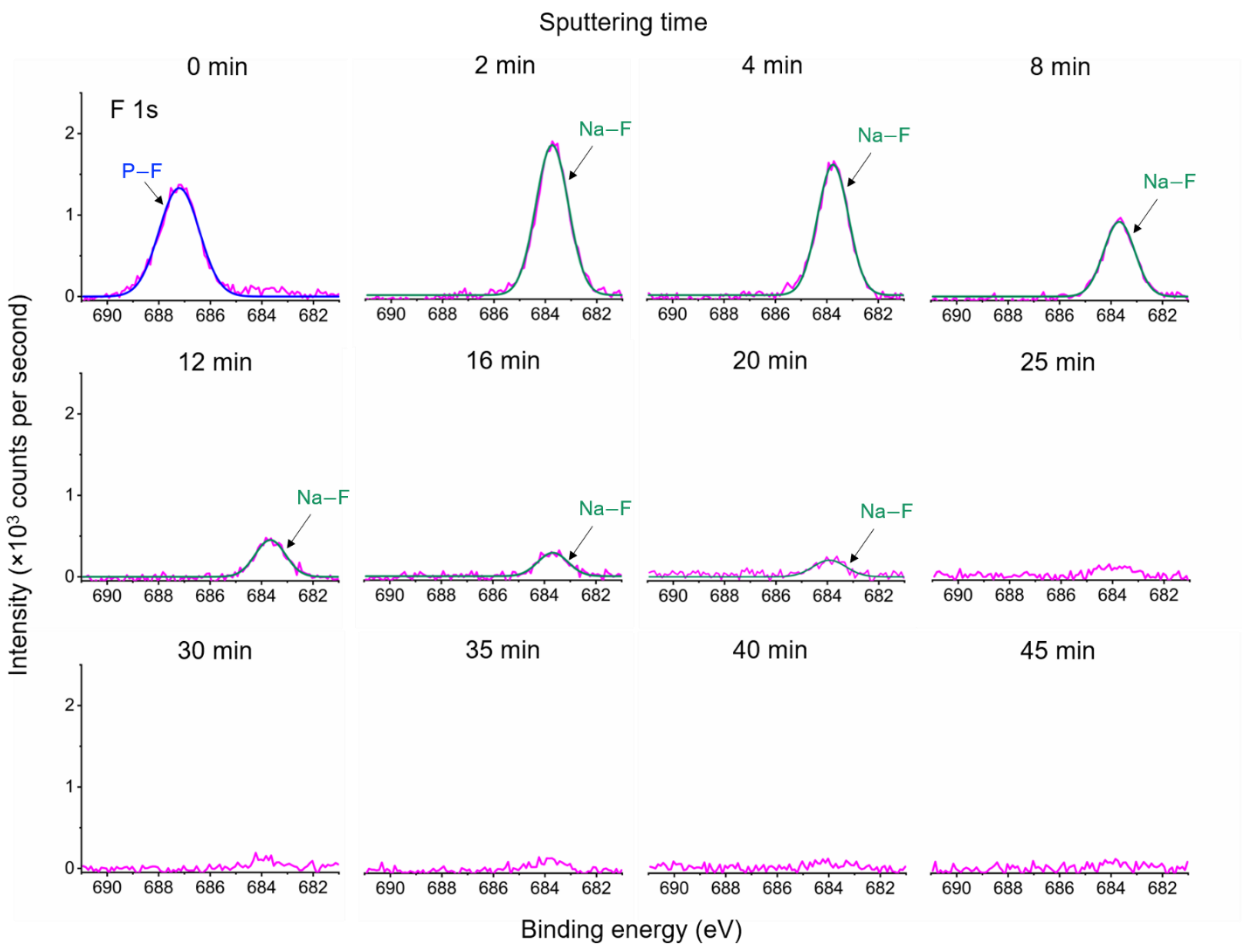

Figure S10c. Details for Figure 3e. XPS characterization of the SEI at deposited state after 100.5 cycles in 1 M $NaPF_6$-diglyme electrolyte. This figure shows the F 1s spectra.

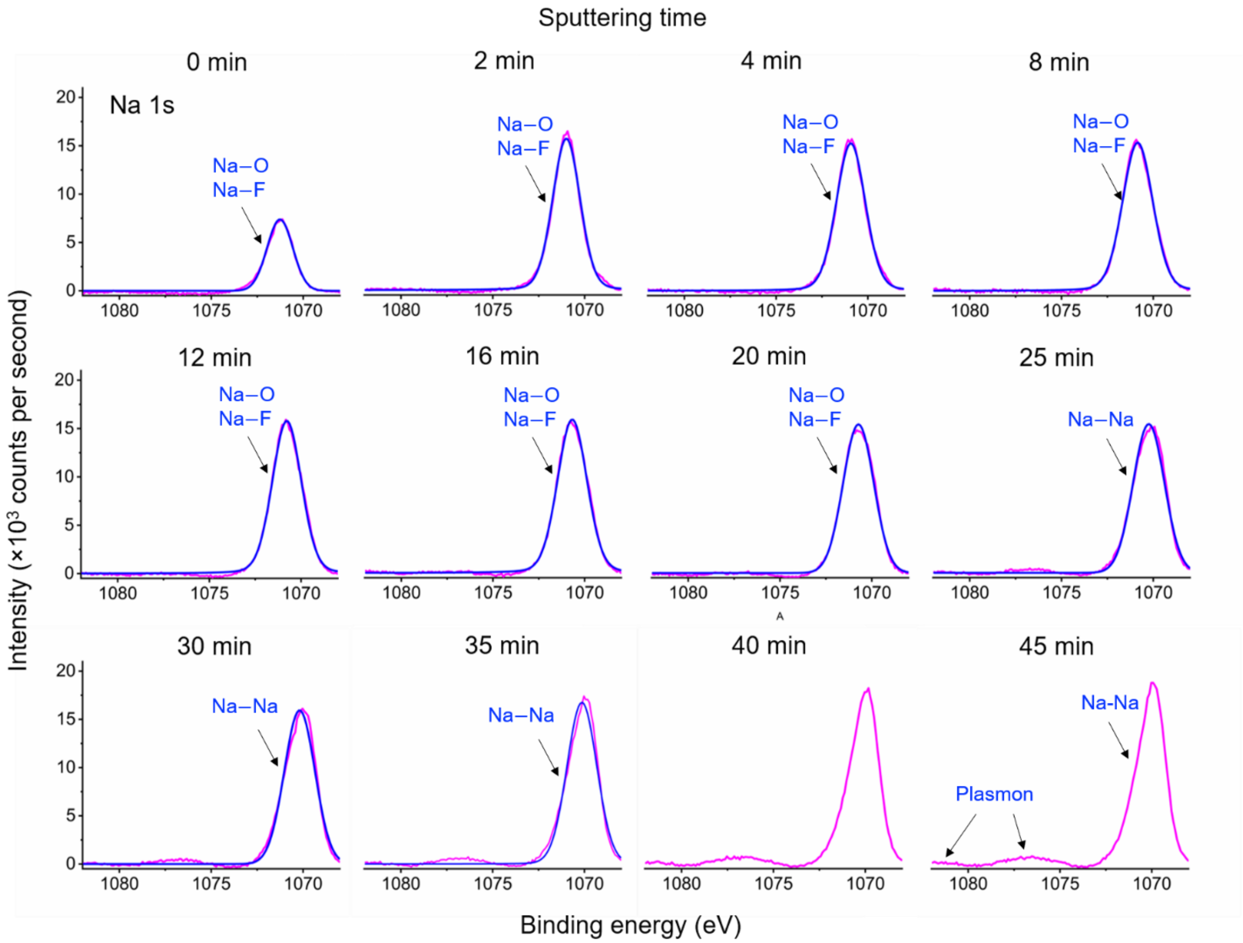

Figure S10d. Details for Figure 3e. XPS characterization of the SEI at deposited state after 100.5 cycles in 1 M $NaPF_6$-diglyme electrolyte. This figure shows the Na 1s spectra.

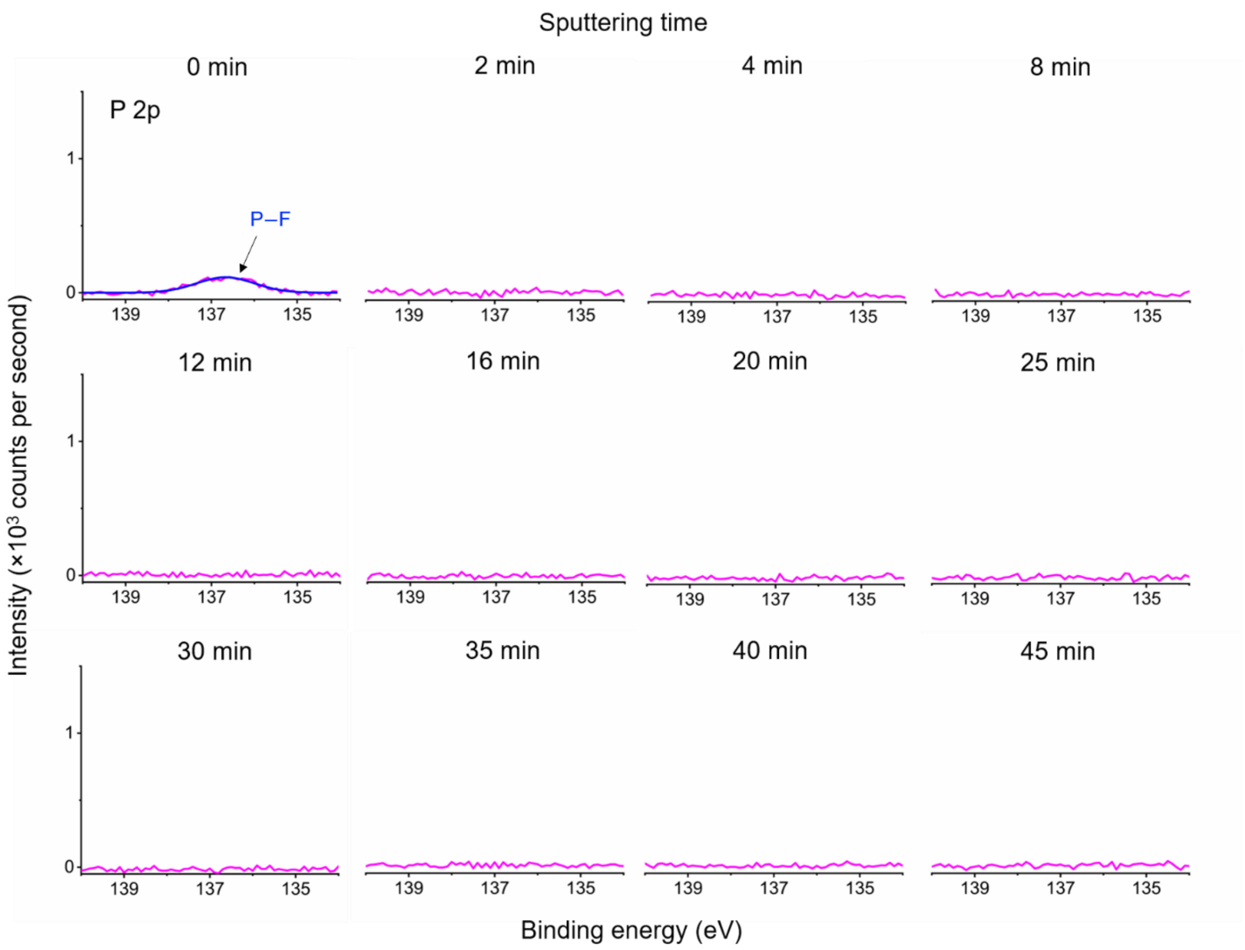

Figure S10e. Details for Figure 3e. XPS characterization of the SEI at deposited state after 100.5 cycles in 1 M $NaPF_6$-diglyme electrolyte. This figure shows the P 2p spectra.

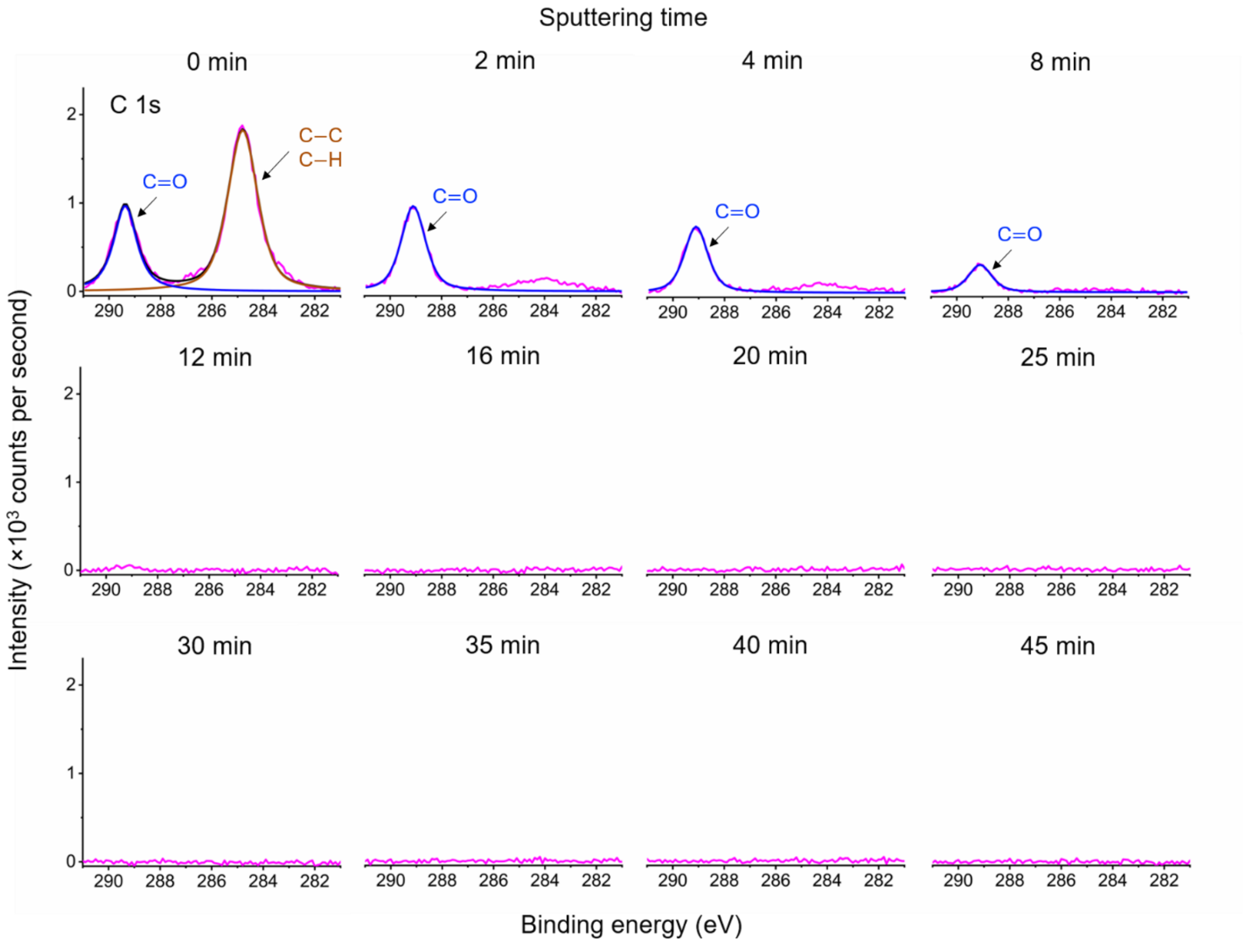

Figure S11a. Details for Figure 3f. XPS characterization of the SEI at deposited state after 10 hours of one-way deposition in 1 M $NaPF_6$-diglyme electrolyte. This figure shows the C 1s spectra.

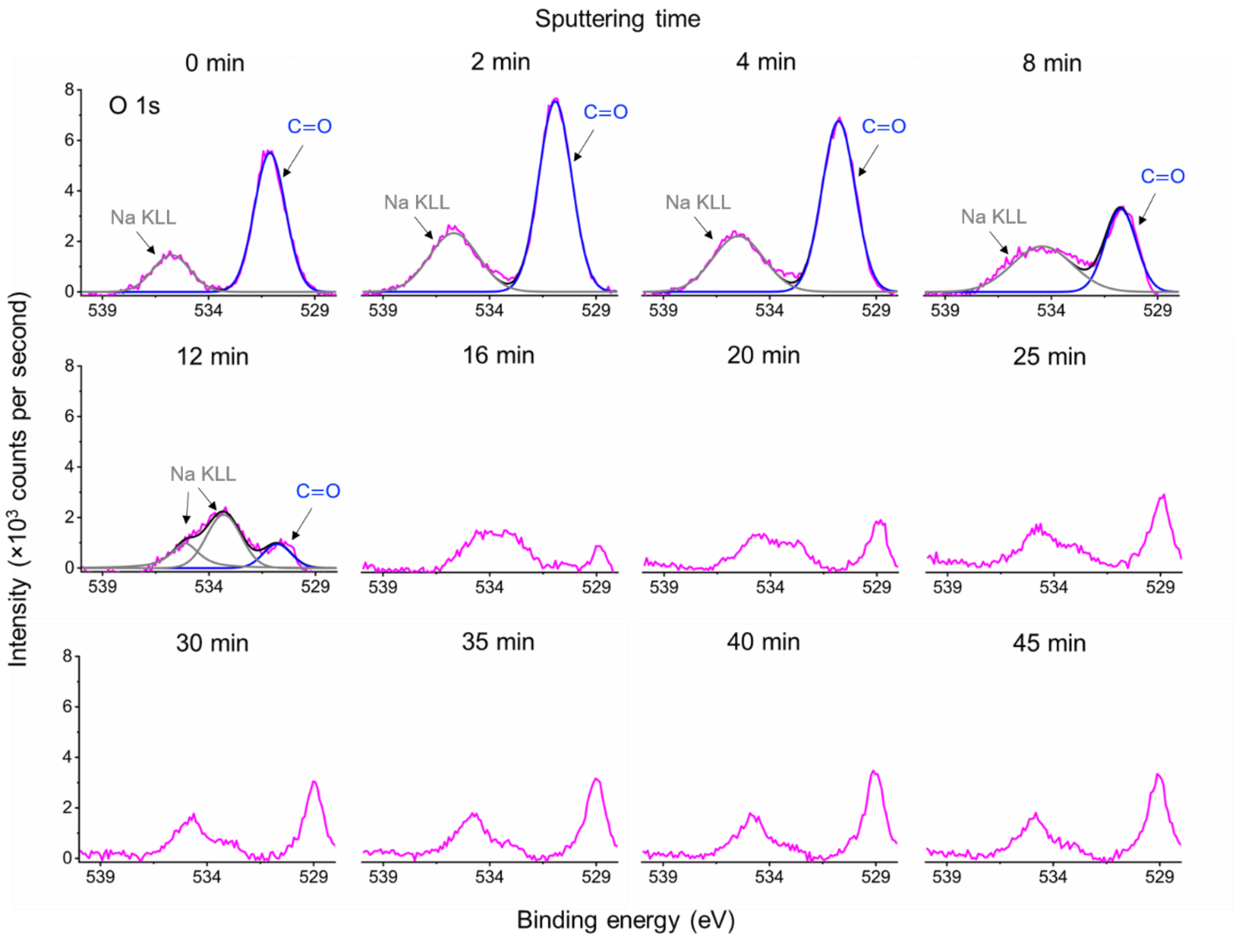

Figure S11b. Details for Figure 3f. XPS characterization of the SEI at deposited state after 10 hours of one-way deposition in 1 M $NaPF_6$-diglyme electrolyte. This figure shows the O 1s spectra.

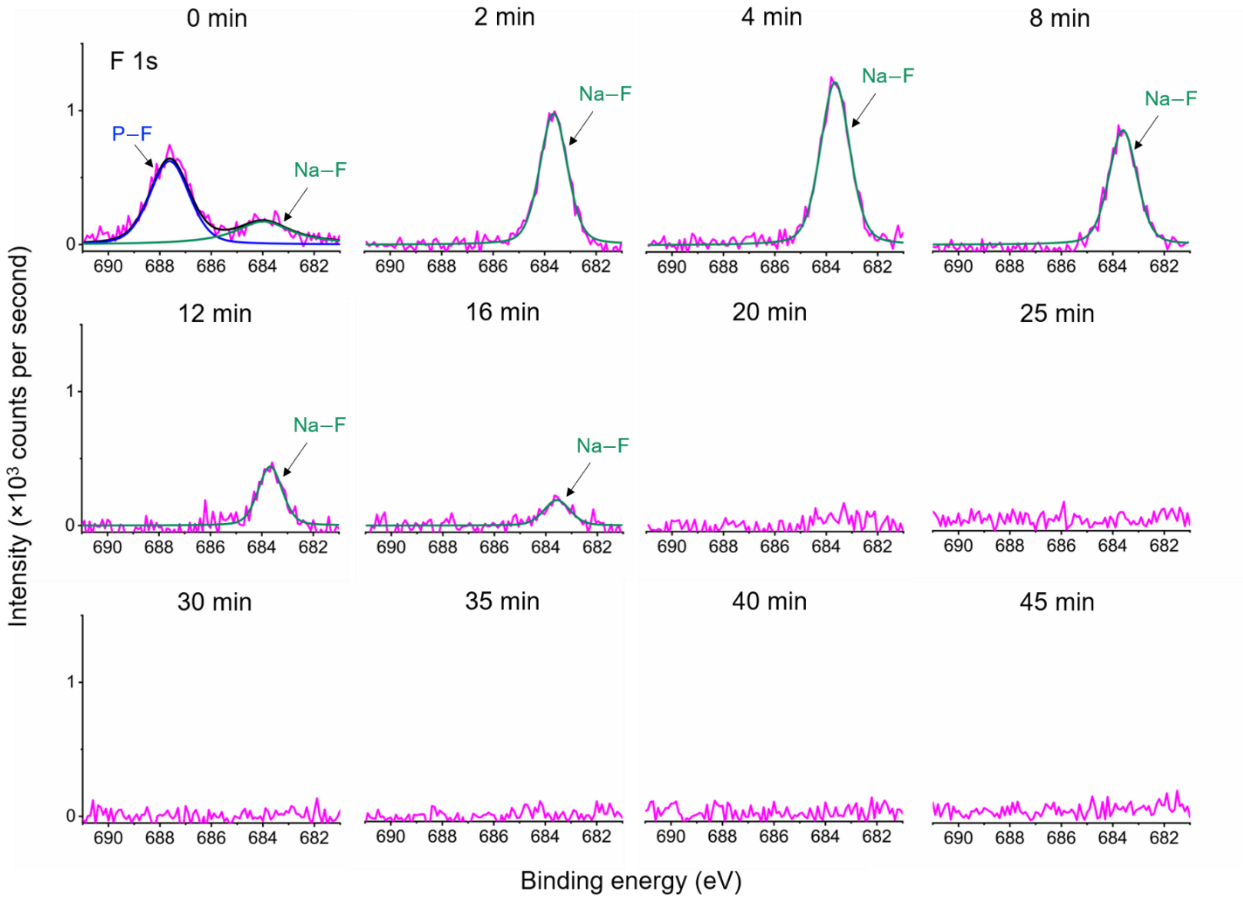

Figure S11c. Details for Figure 3f. XPS characterization of the SEI at deposited state after 10 hours of one-way deposition in 1 M $NaPF_6$-diglyme electrolyte. This figure shows the F 1s spectra.

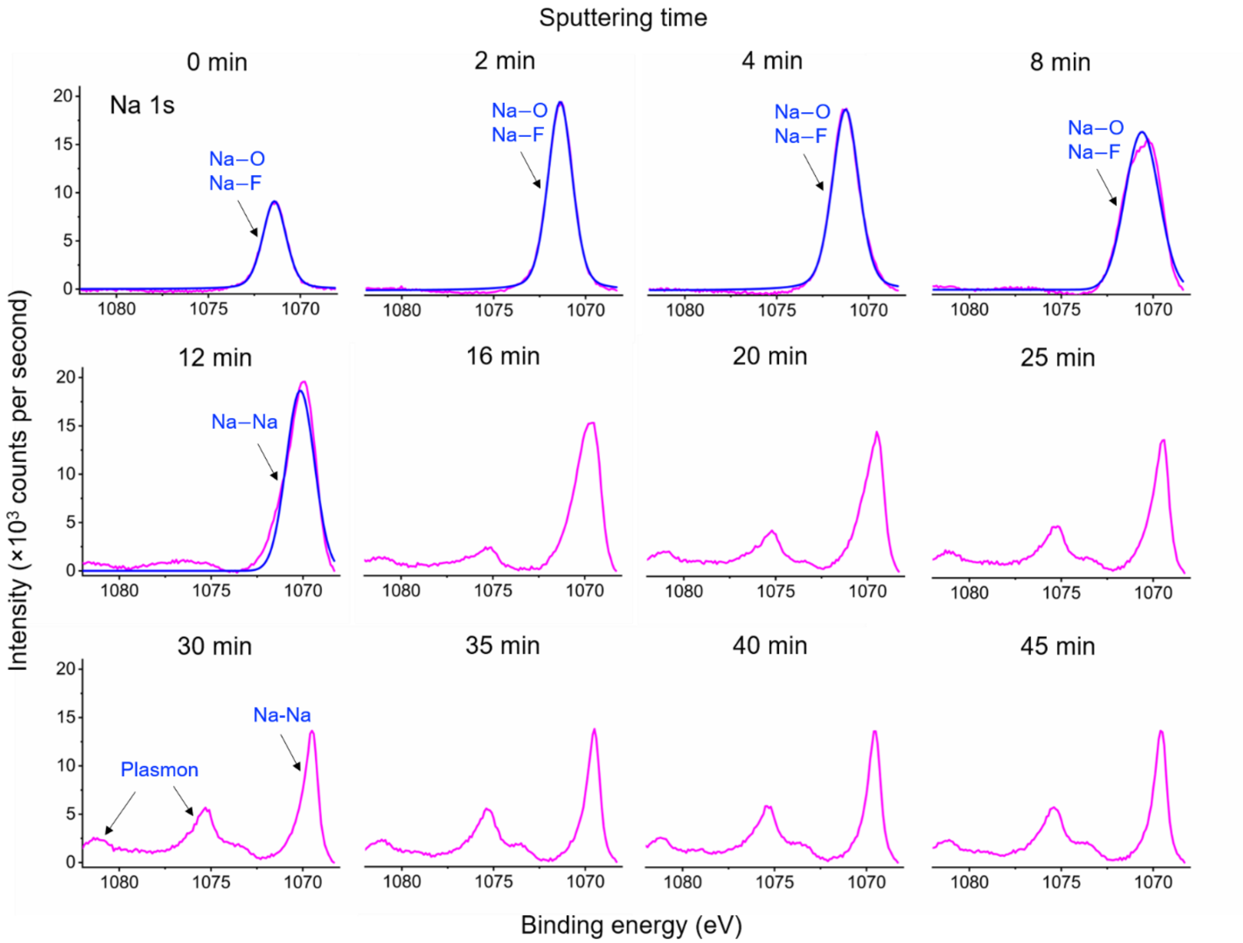

Figure S11d. Details for Figure 3f. XPS characterization of the SEI at deposited state after 10 hours of one-way deposition in 1 M $NaPF_6$-diglyme electrolyte. This figure shows the Na 1s spectra.

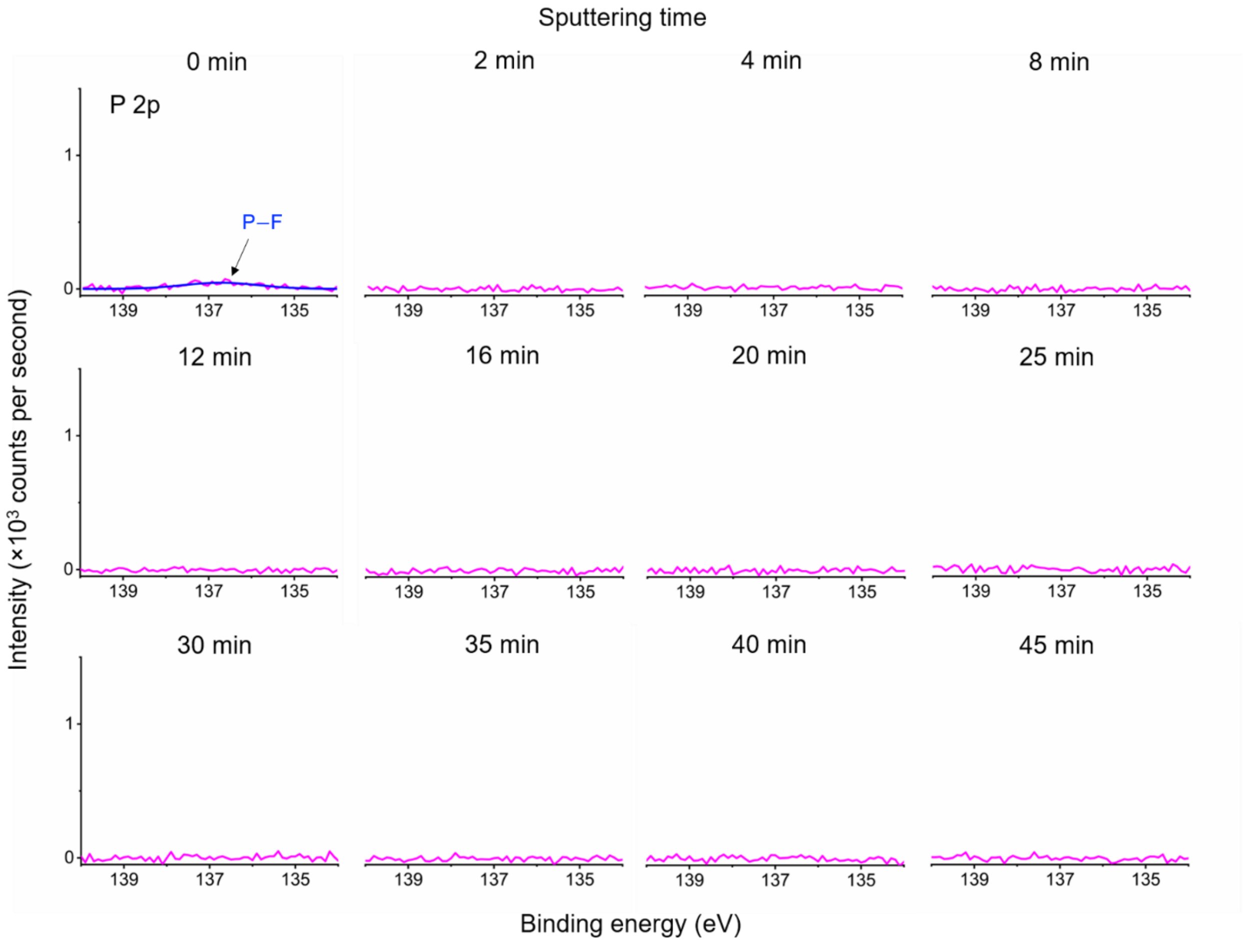

Figure S11e. Details for Figure 3f. XPS characterization of the SEI at deposited state after 10 hours of one-way deposition in 1 M $NaPF_6$-diglyme electrolyte. This figure shows the P 2p spectra.

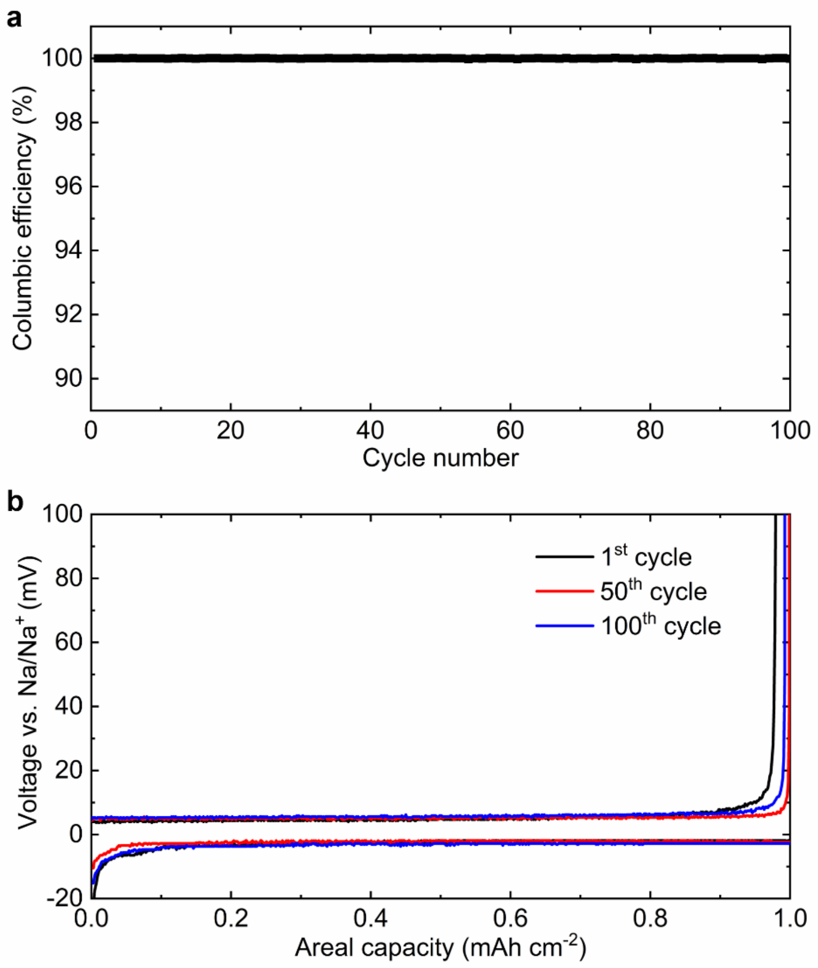

Figure S12. The Coulombic efficiency a) and the voltage response b) cycled at 0.5 mA cm^{-2} and 1 mAh cm^{-2} using 1 M $NaPF_6$-diglyme electrolyte in Cu|Na half cell.

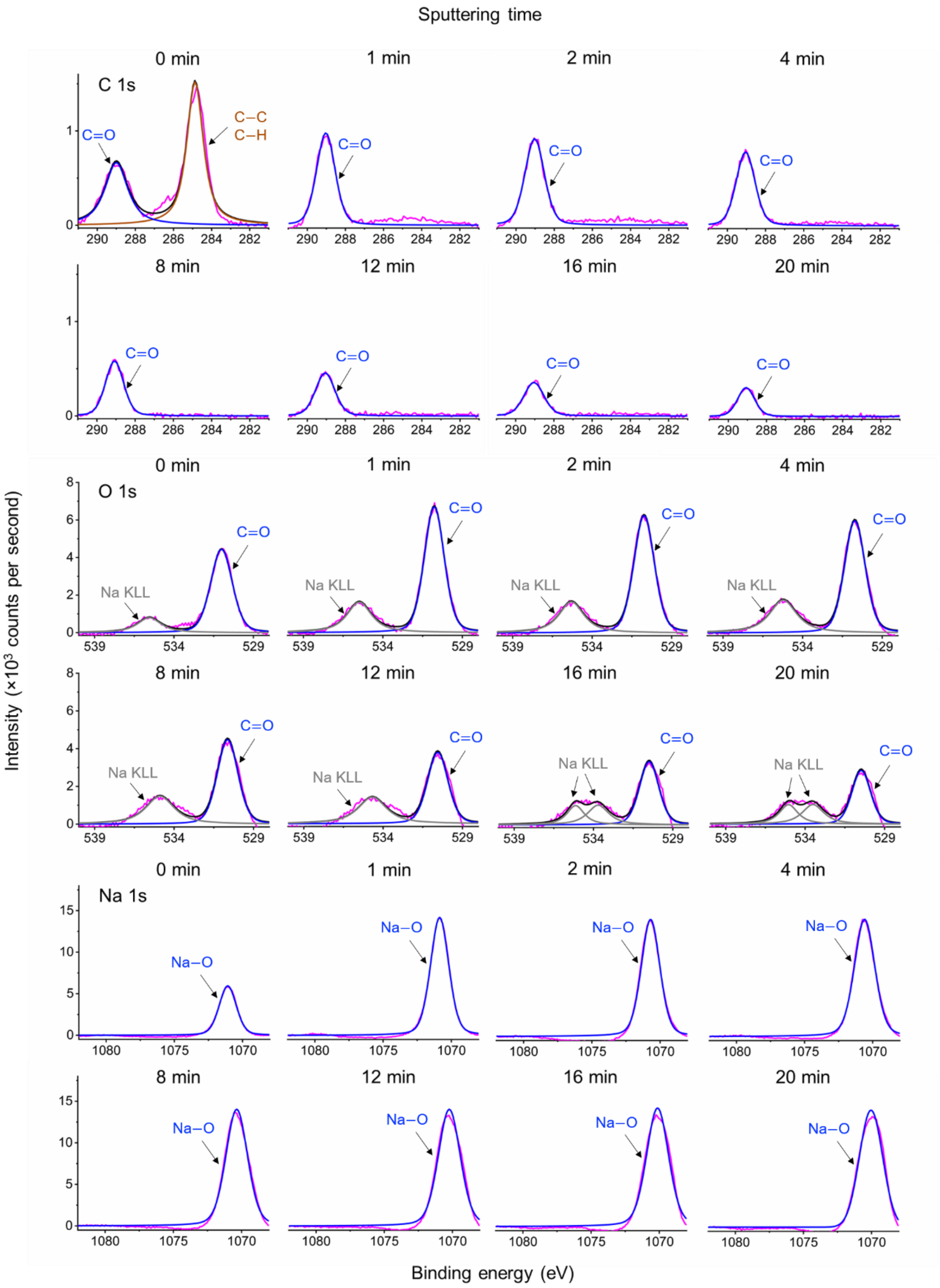

Figure S13. XPS characterization of the surface of a piece of fresh Na.

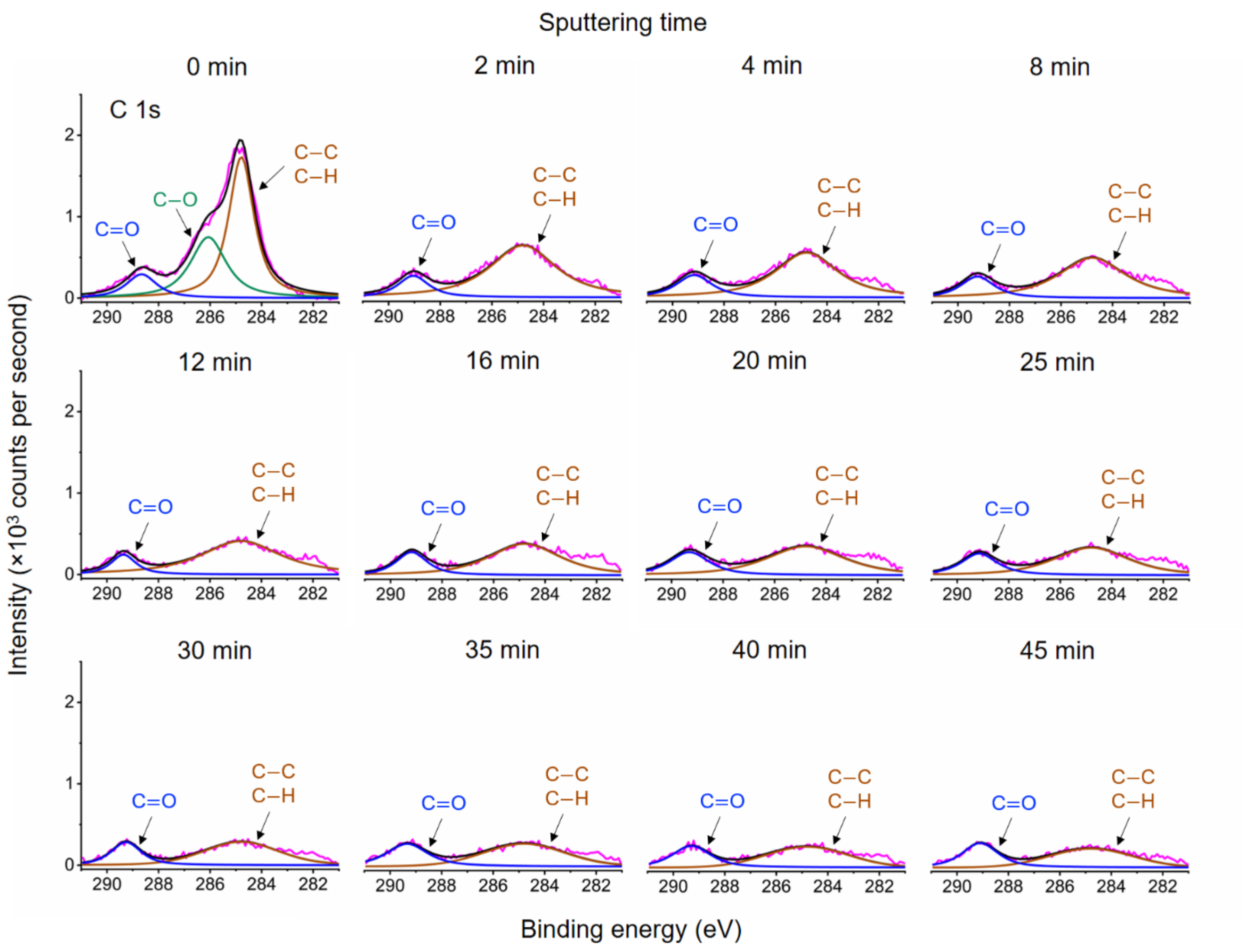

Figure S14a. XPS characterization of the SEI at deposited state after completing the first half cycle of deposition in 1 M $NaClO_4$-diglyme electrolyte. This figure shows the C 1s spectra.

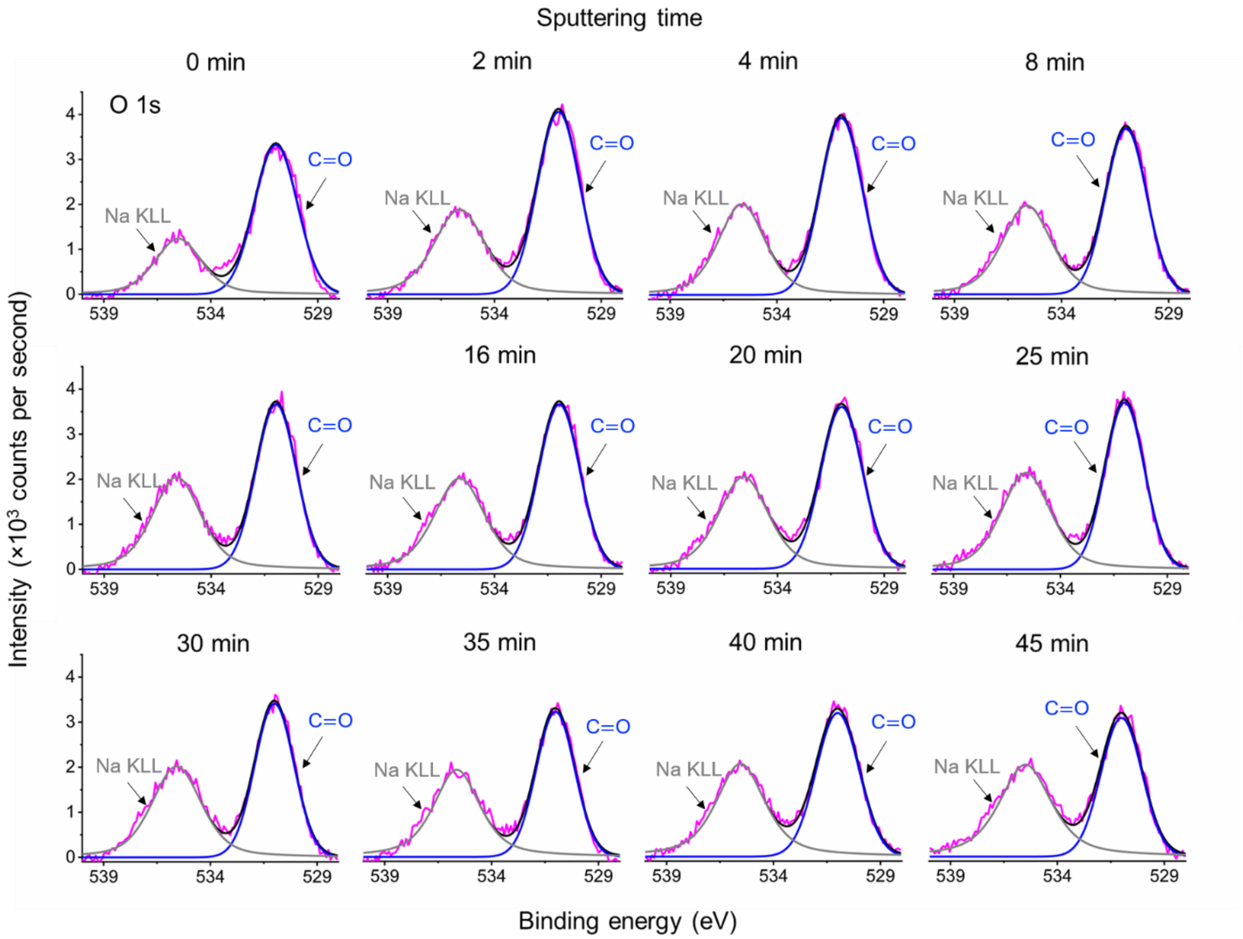

Figure S14b. XPS characterization of the SEI at deposited state after completing the first half cycle of deposition in 1 M $NaClO_4$-diglyme electrolyte. This figure shows the O 1s spectra.

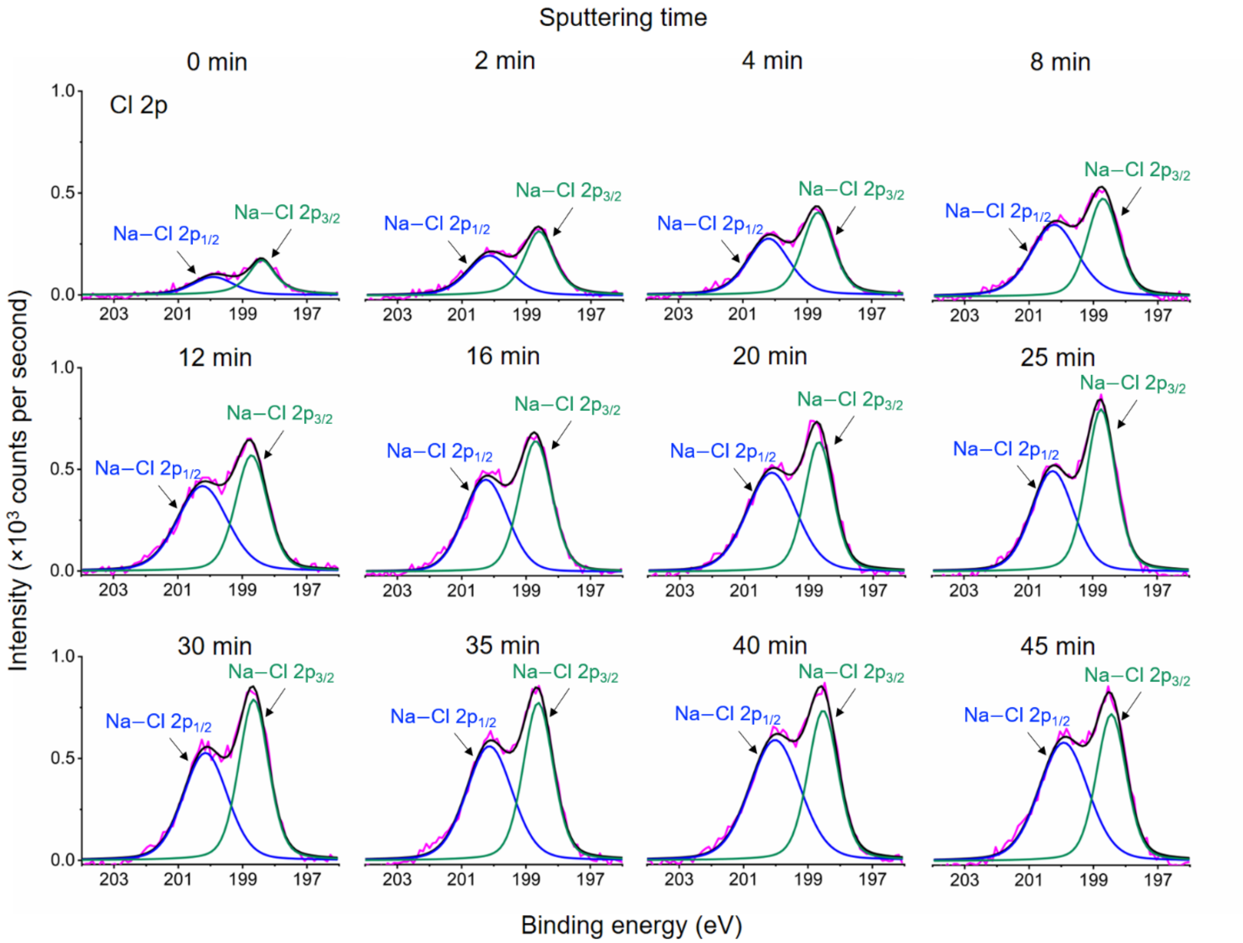

Figure S14c. XPS characterization of the SEI at deposited state after completing the first half cycle of deposition in 1 M $NaClO_4$-diglyme electrolyte. This figure shows the Cl 2p spectra.

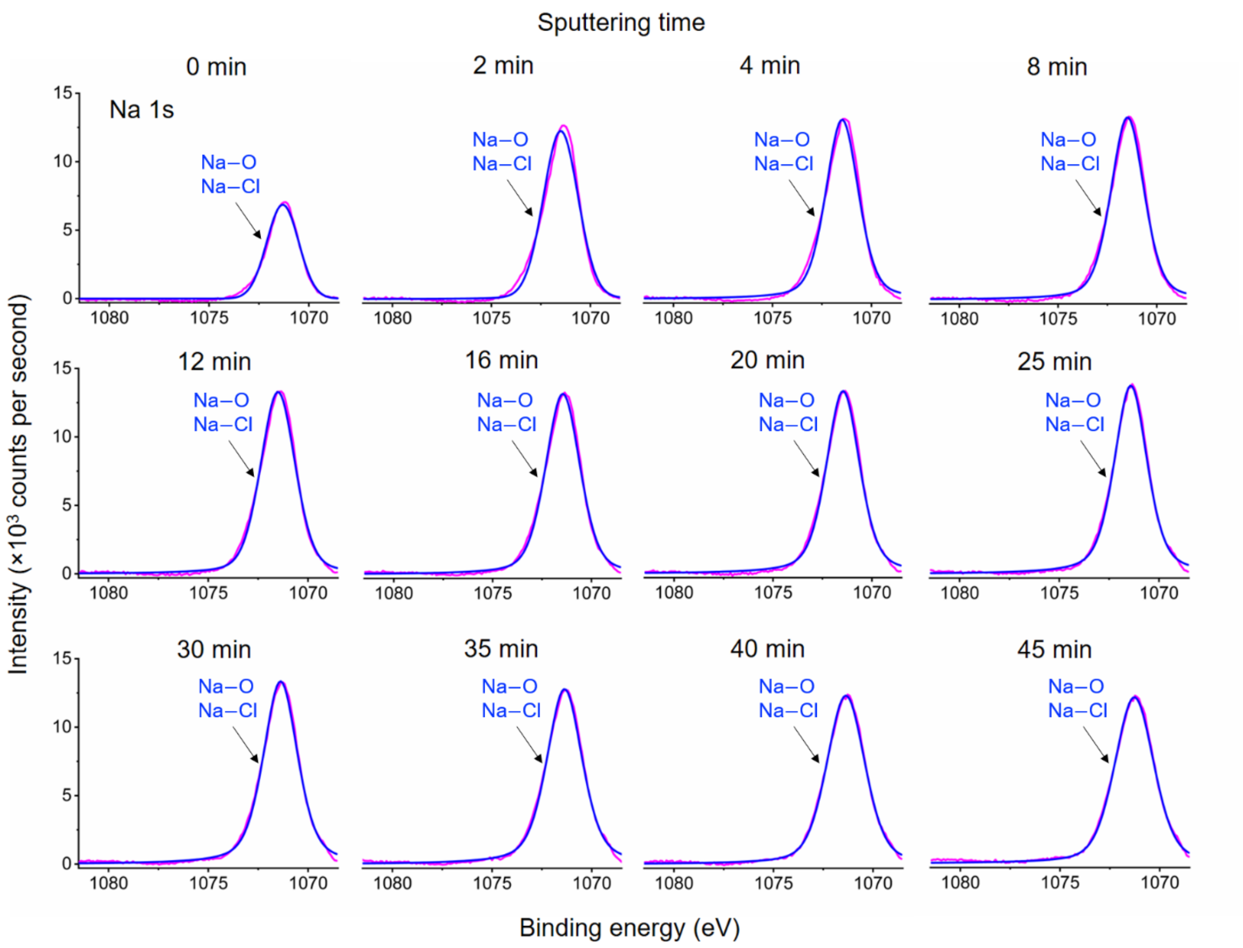

Figure S14d. XPS characterization of the SEI at deposited state after completing the first half cycle of deposition in 1 M $NaClO_4$-diglyme electrolyte. This figure shows the Na 1s spectra.

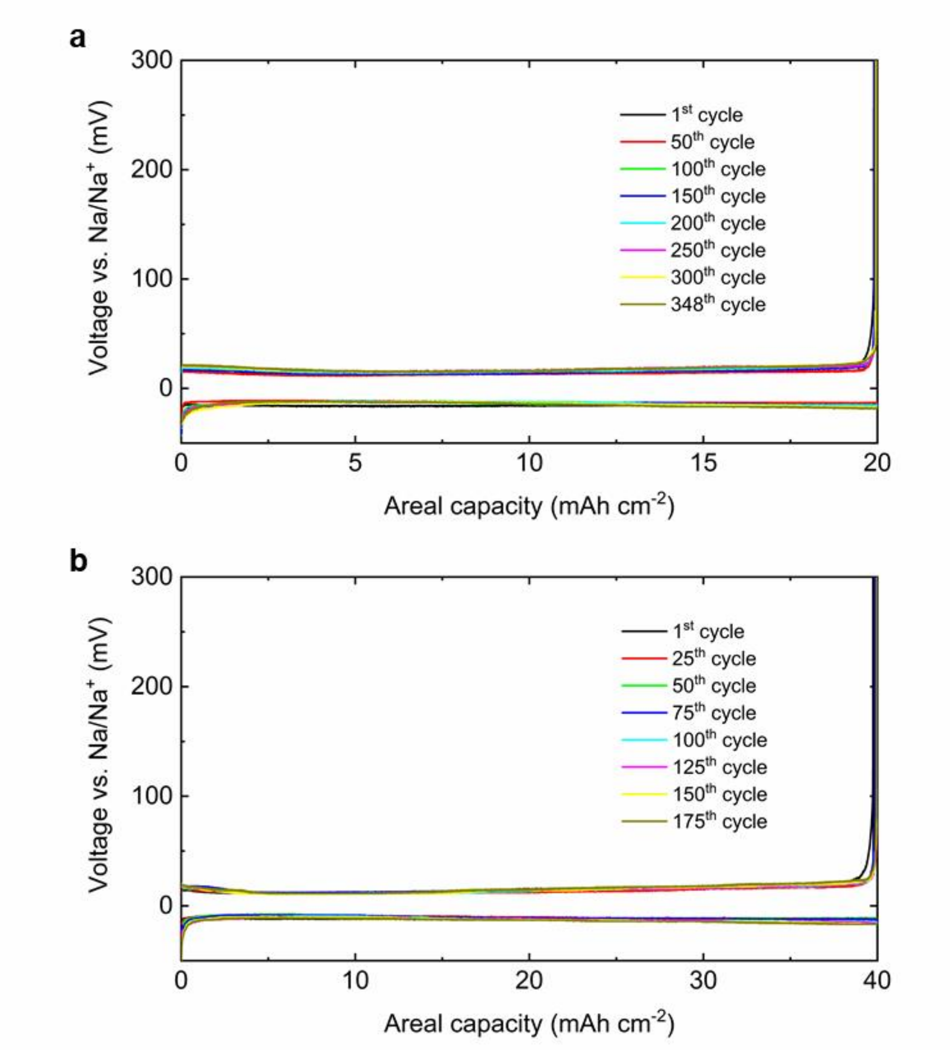

Figure S15. The voltage responses cycled under a) 20 mAh cm^{-2} and b) 40 mAh cm^{-2} at 2mA cm^{-2} using 1 M $NaPF_6$-diglyme electrolyte in Cu|Na half cell.

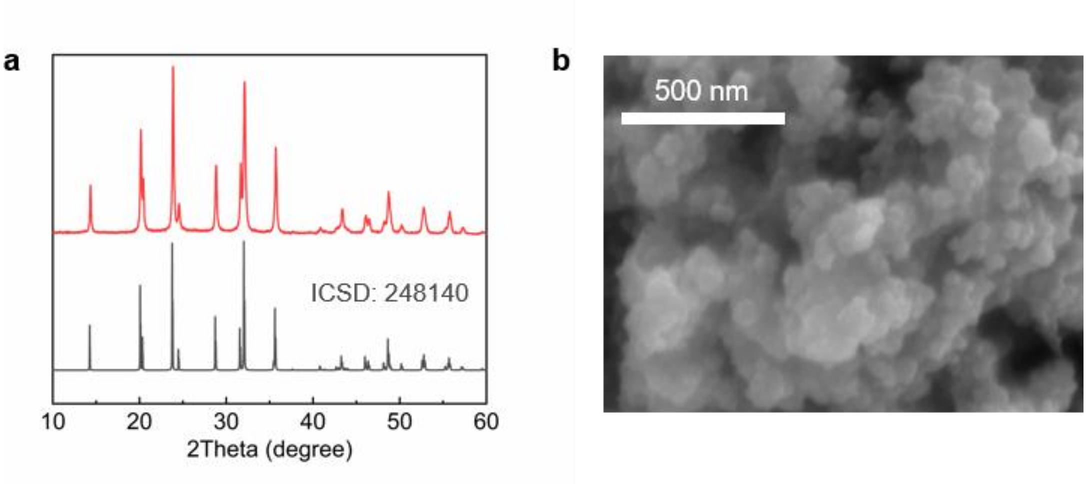

Figure S16. XRD a) and SEM b) characterization for the $Na_3V_2(PO_4)_3$ (NVP) nanomaterial.

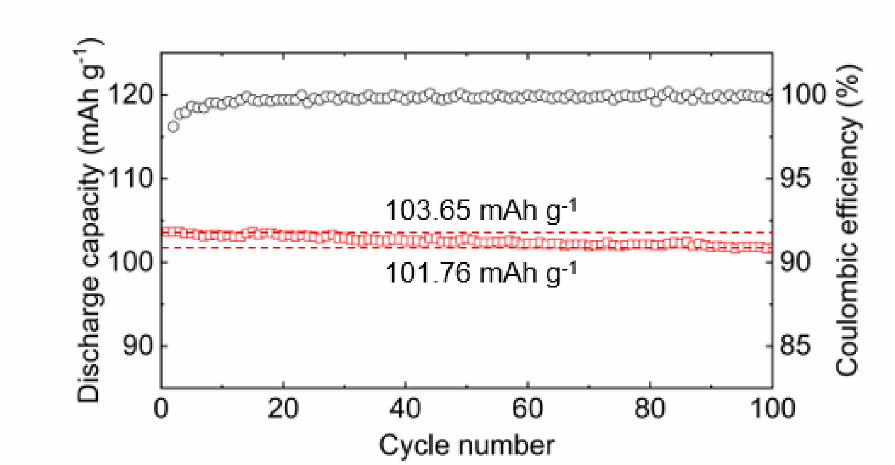

Figure S17. Discharge capacity and coulombic efficiency of the Na|NVP half cell with excess Na, during galvanostatic cycling at 2C. The capacity retention rate per cycle is 99.98%.

a

b

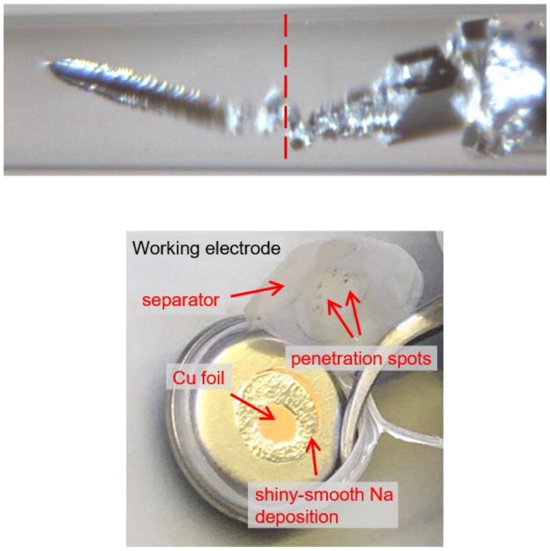

Figure S18. a) The snapshot showing the transition of growth mechanism at an over-limiting current density. Red dashed line indicates the Sand's time, at which the transition happened. b) The optical image of an opened cell after penetration.

References

[1] J. F. Moulder, W. F. Stickle, P. E. Sobol, K. D. Bomben, *Handbook of X-Ray Photoelectron Spectroscopy: A Reference Book of Standard Spectra for Identification and Interpretation of XPS Data*, **1992**.

[2] G. P. López, D. G. Castner, B. D. Ratner, *Surface and Interface Analysis* **1991**, *17*, 267.

[3] Z. W. Seh, J. Sun, Y. Sun, Y. Cui, *ACS Central Science* **2015**, *1*, 449.

[4] A. v. Shchukarev, D. v. Korolkov, *Central European Journal of Chemistry* **2004**, *2*, 347.

[5] P. M. L. Le, T. D. Vo, H. Pan, Y. Jin, Y. He, X. Cao, H. v. Nguyen, M. H. Engelhard, C. Wang, J. Xiao, J. G. Zhang, *Advanced Functional Materials* **2020**, *30*, 1.

[6] J. Zhang, D. W. Wang, W. Lv, S. Zhang, Q. Liang, D. Zheng, F. Kang, Q. H. Yang, *Energy and Environmental Science* **2017**, *10*, 370.

[7] A. Barrie, F. J. Street, *Journal of Electron Spectroscopy and Related Phenomena* **1975**, *7*, 1.

[8] J. C. Dupin, D. Gonbeau, P. Vinatier, A. Levasseur, *Physical Chemistry Chemical Physics* **2000**, *2*, 1319.
